# Experimentally Determined Hansen Solubility Parameters of Biobased and Biodegradable Polyesters

**DOI:** 10.1021/acssuschemeng.3c07284

**Published:** 2024-02-01

**Authors:** Kush G. Patel, Ryan K. Maynard, Lawrence S. Ferguson, Michael L. Broich, Joshua C. Bledsoe, Caitlin C. Wood, Grant H. Crane, Jessica A. Bramhall, Jonathan M. Rust, Amanda Williams-Rhaesa, Jason J. Locklin

**Affiliations:** † School of Chemical, Materials, and Biomedical Engineering, College of Engineering, University of Georgia, 597 D.W. Brooks Dr., Athens, Georgia 30602, United States; ‡ Department of Chemistry, Franklin College of Arts and Sciences, University of Georgia, 140 Cedar Street, Athens, Georgia 30602, United States; § New Materials Institute, University of Georgia, 220 Riverbend R., Athens, Georgia 30602, United States

**Keywords:** Hansen solubility parameters, biobased plastics, biodegradable plastics, solubility, biobased plasticizers, plasticizer compatibility

## Abstract

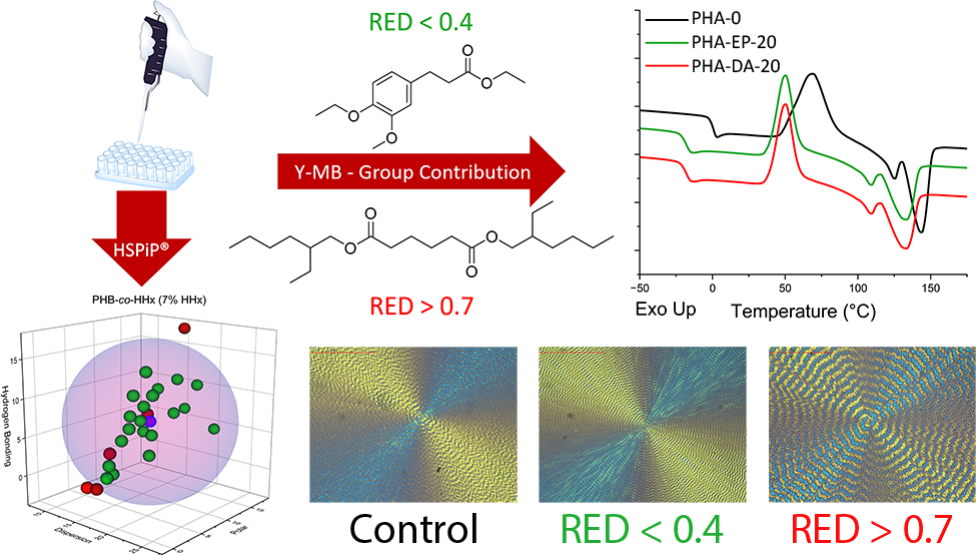

Hansen solubility parameters (HSP) of 15 commercially relevant biobased and biodegradable polyesters were experimentally determined by applying a novel approach to the classic solubility study method. In this approach, the extent of swelling in polymer films was determined using a simple equation based on the mass difference between swollen and nonswollen film samples to obtain normalized solvent uptake (***N***). Using ***N*** and HSPiP software, highly accurate HSP values were obtained for all 15 polyesters. Qualitative evaluation of the HSP values was conducted by predicting the miscibility of poly(3-hydroxybutyrate-*co*-3-hydroxyhexanoate) (PHB-*co*-HHx, 7 mol % HHx) and poly(lactic acid) (PLA) with a novel lignin-based plasticizer (ethyl 3-(4-ethoxy-3-methoxyphenyl)propanoate, EP) with a relative energy difference (RED) less than 0.4. Additionally, an HSP-predicted plasticizer (di(2-ethylhexyl) adipate, DA) with a larger RED (>0.7) was used to demonstrate the effects of less-miscible additives. Plasticized samples were analyzed by differential scanning calorimetry and polarized optical microscopy (POM) to determine the *T*_g_ depression, with EP showing linear *T*_g_ depression up to 50% plasticizer loading, whereas DA shows minimal *T*_g_ depression past 10% loading. Further analysis by POM reveals that the DA phase separates from both polymers at loadings as low as 2.5% (PHB-*co*-HHx, 7 mol % HHx) and 5% (PLA), while the EP phase separates at a much higher loading of 50% (PHB-*co*-HHx, 7 mol% HHx) and 30% (PLA).

## Abstract 2

Solubility parameters are commonly used to determine compatibility between polymers and additives. This article reports experimentally determined Hansen solubility parameters of biobased and biodegradable plastics. Additionally, the solubility parameters were used to develop a novel lignin-derived plasticizer, which was blended with selected polyesters from this study. Blends of the new plasticizer were analyzed by differential scanning calorimetry and polarized optical microscopy to determine changes in glass transition temperature and additive miscibility.

## Introduction

Hansen solubility parameters (HSP) provide an empirical guide to compatibility between polymers and solvents. Initially, HSP were developed primarily for use in the paint and coating industry.^1,2^ Today, HSP of polymers contribute significantly to formulation development in many industries, ranging from food packaging to pharmaceutical formulations.^1,3,4^ For commodity plastics, HSP is used to predict plasticizer compatibility, chemical resistance, and permeation rates. The predictive powers of HSP have also been extended to insoluble materials such as fillers, pigments, and fibers.^1,2,5^

HSP values for commodity and specialty petrochemical-based polymers such as polyolefins, polyamides, polyesters, etc., have been reported.^1^ However, the use of these plastics, especially for single-use applications, has had a significant impact on the environment as almost none of these materials are biodegradable, leading to their accumulation in landfills or leakage into the natural environment.^6,7^ To address this growing concern, industrial and academic research has focused on developing novel biobased and biodegradable polymers to replace commodity plastics, especially in packaging and single-use applications.

Biobased plastics are synthesized using monomers derived from sustainable and renewable feedstocks via processes such as fermentation and ozonolysis.^8−12^ Plastics such as poly(ethylene furanoate) (PEF), cellulose esters, and biobased poly(ethylene) (bio-PE) are produced using sustainable and renewable resources.^9−11^ Biodegradable plastics are degradable plastics in which the degradation results from the action of naturally occurring microorganisms such as bacteria, fungi, and algae.^9,13−15^ The process of biodegradation depends on the polymer and environmental factors, such as moisture, temperature, inoculum, and microbial load. For practical purposes, regional and global standards have been established to define guidelines for biodegradation in various environmental conditions within reasonable timeframes.^9,16−18^ The biodegradation of polymers is a complex topic, and biodegradability is sensitive to the receiving environment (soil, landfill, marine, etc.). Detailed information on the biodegradability of polyesters and different standards used to measure biodegradation is provided in the Supporting Information (Tables S1 and S2).

Bioplastics face challenges as drop in replacements for commodity plastics due to differences in their mechanical properties and processing parameters.^9,10,13−15,17,19−25^ For example, poly(lactic acid) (PLA) has a higher elastic modulus compared to polypropylene, acrylonitrile butadiene styrene, or polyamide, but is also more brittle and has a relatively low heat deflection temperature.^26^ Low *T*_g_ aliphatic polyesters such as polyhydroxyalkanoates (PHAs) can potentially replace polyolefins but lag behind due to their processing limitations, relative brittleness, and lower chemical resistance. Extensive research has been conducted to improve the processing and properties of biopolymers to replace commodity plastics.^8−10,12,18,23,25,27−30^

Most commercial plastic resins and products are formulated with additives ranging from processing aids to plasticizers to reinforcing agents.^31,32^ The discovery of these additives and the exact amounts needed were the product of decades of research and development from academia and industry. Over the same course of time, processing equipment for specific plastics was also developed along with optimal processing conditions. In recent years, concerns have been raised over the adverse effects of many of these additives on human and ecological health. Among them, perfluoroalkyl and polyfluoroalkyl substances (PFAS), bisphenol A (BPA), and phthalate plasticizers have received widespread scrutiny with multiple class action lawsuits and legislative bans. However, finding biologically inert as well as biobased additives that can replace these materials has been a challenging pursuit.^10,11,23−25,33^ Accurate HSP values can significantly expedite the screening process for existing additives as well as open new doors for the development of novel biobased additives such as plasticizers, pigments, stabilizers, reinforcing agents, etc.^1,2,5^

HSP can be determined theoretically or experimentally. Theoretical HSP values are calculated by using group contribution methods. A molecule can be broken down into several subgroups, and each subgroup has some HSP contribution. The contributions are determined by correlating mathematical models with experimentally generated data.^1,34,35^ A molecule such as ethyl acetate (CH_3_CO_2_CH_2_CH_3_) can be broken down into three subgroups: –COO (ester), –CH_3_, and –CH_2_CH_3_. By adding the contributions of these subgroups relative to their presence in a molecule, theoretical HSP values can be determined. Many researchers have published their calculation methods and HSP values associated with subgroups for predicting HSP values. Methods by Hoy, Fedors, Van Krevelan, Stefanis-Panayiotou, Yamamoto, and others have proven their reliability for predicting small-molecule HSP.^1,34,35^ However, for larger molecules such as polymers, these theoretical methods tend to fall short, and experimental data is preferred.^1,36^

Experimental determination of polymer HSP is done using indirect methods, where solvents of known HSP values are mixed with polymers. Experimental methods include solubility and swellability studies, intrinsic viscosity, and melting point depression. Solubility/swellability studies are conducted by soaking a compound in various solvents with known HSP values and equating the degree of solvation to solvent compatibility. The compatibility of a polymer and solvent can also be studied by measuring the intrinsic viscosity of the solution. Viscosity measurements of polymer solutions in various solvents help to determine the amount of polymer dissolved in a solvent. Solutions with higher intrinsic viscosity have more polymer dissolved, which indicates better compatibility. Melting point depression is another method used to determine solvent miscibility, where the temperature reduction is dependent on the degree of swelling and the inherent properties of the solvent.^1,4^

In this study, we have experimentally determined the HSP values for poly(3-hydroxybutyrate-*co*-3-hydroxyhexanoate) (PHB-*co*-HHx, four different comonomer contents), poly(3-hydroxybutyrate-*co*-4-hydroxybutyrate) (PHB-*co*-4HB), PLA (three different commercial grades), poly(butylene succinate) (PBS), poly(butylene succinate-*co*-butylene adipate) (PBSA), poly(butylene adipate-*co*-butylene terephthalate) (PBAT), poly(caprolactone), poly(trimethylene terephthalate) (PTT), and PEF. Additionally, a novel lignin-derived plasticizer and an incompatible plasticizer determined by HSPiP software were used to verify the quality of the HSP values produced.

## Materials and Experimental Methods

### Materials

#### Polymers

PLA resins Ingeo 4032D (1.5 mol % d-lactide)^14^ and Ingeo 4060D (12 mol % d-lactide)^14^ were purchased from Natureworks LLC. Ingeo 2500HP (0.5 mol % d-lactide)^37^ and Ingeo 4950D (45 mol % d-lactide) were donated by Natureworks LLC. PBS (BioPBS FZ91PM) and PBSA (BioPBS FD92PM) (20 mol % adipate)^38^ were purchased from Mitsubishi Chemical Corporation. PBAT (Ecoflex F Blend C1200) was procured from BASF. PCL (Capa 6800D) was purchased from Ingevity. PTT (Sorona Bright) was donated by Covation Biomaterials. Four grades of PHB-*co*-HHx (0 mol % HHx, 7 mol % HHx, 12 mol % HHx, and 18 mol % HHx) were provided by the New Materials Institute at the University of Georgia. PHACT A1000 (PHB-*co*-4HB) (>30 mol % 4HB, reported by the manufacturer) was obtained from CJ Bio America Inc. PEF was synthesized for this study. All polymers were dried under vacuum (>40 °C, according to manufacturer guidelines) and used without further purification.

#### Solvents and Reagents

All reagents were purchased from commercial suppliers and used as received, unless otherwise noted.

### Synthesis

Detailed synthetic procedures and characterization for compounds dimethyl-2,5-dicarboxylate, PEF, and ethyl 3-(4-ethoxy-3-methoxyphenyl)propanoate (EP) are provided in the SI.

### Characterization

#### Differential Scanning Calorimetry

Differential scanning calorimetry (DSC) analysis was conducted on a Discovery DSC 250 (TA Instruments, USA) differential scanning calorimeter equipped with an RCS 90 cooling system (TA Instruments, USA) under a 50 mL/min nitrogen purge. The samples (2–7 mg) were enclosed in aluminum T-zero pans. Samples were first heated to peak temperatures ranging from 200 to 260 °C at 10 °C/min to erase thermal history. Samples were subsequently cooled to −70 °C at 200 °C/min, followed by a second heating step to peak temperatures at 10 °C/min. Peak heating temperatures were chosen to include melting transitions without thermal degradation. Percent crystallinity was calculated using heat of fusion (Δ*H*_0_) values obtained from literature (Table S3).

#### Nuclear Magnetic Resonance

^1^H NMR spectra were recorded at room temperature with a Bruker Neo 600 MHz (USA) at a concentration of 10 mg/mL. A peak shift in parts per million (ppm) was reported relative to the signal of chloroform at 7.26 ppm.

#### Gel Permeation Chromatography

Molecular weight and molecular weight distribution analyses of samples were conducted by using a Malvern OMNISEC RESOLVE gel permeation chromatography (GPC) system (Malvern, UK) equipped with a refractometer, right-angle and low-angle light scattering detector (RALS/LALS), and viscometer. Samples of 1 mg/mL concentration were prepared in HPLC-grade chloroform and filtered through a 0.2 μm PTFE filter to remove trace contaminants. The GPC columns (Viscotek T Series; T3000, T4000, and T6000) regulated at 35 °C were used to facilitate separation with HPLC-grade chloroform as the eluent set at a flow rate of 1 mL/min. The collected data were processed and analyzed using OMISEC-v11.10 software.

### Melt Pressing Polyester Films

Polyester films were melt-pressed using a Carver model 4386 hydraulic press system (USA). The samples were placed between heated platens for 30 s at 1–4 tons of pressure 10 °C above their *T*_m_. The films were removed from the press and aged for 7 days at room temperature before testing.

### Solubility Study for Polymers

The solubility study followed a modified method adapted from Zellers et al.^36^ A circular punch was used to produce 12.7 mm (1/2 in.) diameter samples from aged polymer films. Each film was submerged in solvent for 5 days at a concentration of 1 mg of polymer/mL solvent. After 5 days, the films were blotted dry using Kimwipes and weighed to measure the swollen film mass (*m*_swollenfilm_). The films were then dried in a vacuum oven set to >40 °C for >24 h and reweighed to obtain dry film mass (*m*_dryfilm_). The mass difference between the swollen and dry film was normalized using the dry film mass and solvent density (*d*_solvent_) to obtain the normalized solvent uptake (***N***) (eq 1).
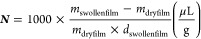
1

The normalized solvent uptake values were used to assign HSP scores from 1 to 6 for each solvent, with 1 being the best solvent (complete dissolution) and 6 being the worst (little to no solvent uptake). The values between 1 and 6 were graded on the degree of solvent uptake, which varied with each polymer. Table S4 contains the grading criteria for each polymer, and Table S5 contains the scores assigned to each polymer/solvent pair. Solvents that scored a 4 or 5 were set as “inside” values for the genetic algorithm in the software (Hansen Solubility Parameters in Practice, HSPiP V5.4.01). The solubility parameters, radius of interaction (*R*_o_), and fit values of each polymer were obtained from HSPiP.

### Novel Plasticizer Discovery and Compatibility

#### In Silico HSP Calculations

The chemical structure of the compatible plasticizer, EP, was transformed using ChemDraw (V21.0.0) into its Simplified Molecular Input Line Entry Syntax (SMILES) code. The SMILES code was used as input for the built-in group contribution method in HSPiP known as Yamamoto molecular break (Y-MB) to compute HSP values of EP.^35^ Compatibility between the polymers and plasticizer was determined using the solvent optimizer function in HSPiP. To show the effects of an incompatible plasticizer, di(2-ethylhexyl) adipate (DA) was identified from the HSPiP plasticizer database.

#### Preparation of POM Samples

Polymer was dissolved with plasticizer in chloroform at a concentration of 40 mg/mL and filtered through a 0.2 μm filter. 250 μL of the polymer solution was drop-cast onto a glass slide and dried under vacuum for 24 h at room temperature for analysis under polarized optical microscopy (POM) and DSC. Percent loadings of 2.5, 5, 10, 15, and 20% were investigated for DA. Percent loadings of 5, 10, 15, 20, 30, and 50% were investigated for EP.

#### Polarized Optical Microscopy

The spherulite morphology of plasticized and unplasticized samples was studied using a Nikon Eclipse LV100N POL (Nikon USA, USA) microscope equipped with a TMHS600 temperature-controlled stage and LINK (ver.1.2.5.1300, Linkam Scientific Instruments, UK). The analyzer and polarizer were both set to 0°, and a 1λ (λ = 530 nm) tint plate was added in the optical path. All camera settings (exposure, shutter, gain, and white balance) were set to adjust automatically for optimal picture quality. To generate spherulites, samples were heated on the stage from room temperature to >40 °C above their melt temperatures at a rate of 150 °C/min and held isothermally for 1 min to erase thermal history. The samples were then cooled to their crystallization temperatures at a rate of 150 °C/min and held isothermally for 60 min.

## Results and Discussion

### Solvent Resistance and Swelling Behavior of Polyesters

Dissolution of a polymer into a solvent is a multistep process which begins with solvent wetting and subsequent diffusion into the polymer matrix that induces swelling. This process can be as quick as a few minutes or can take days depending on the polymer’s molecular weight, solvent compatibility, and temperature. The modes of solvent diffusion can also vary due to differences in *T*_g_, polymer crystallinity, and cross-linking.^39^ Because each of these factors impacts solvation and swelling behavior differently, the extent of solubility is different for each polymer, and the scoring regime for describing good and bad solvents is modified to reflect that.

The changes in solubility due to crystallinity are readily noticeable in copolymers of PHB-*co*-HHx and PHB-*co*-4HB, where increasing HHx and 4HB contents reduces the crystallinity (Figure S1 and Table 1) due to their exclusion from the crystal lattice.^20−22^ This results in PHAs with higher HHx and 4HB contents being more susceptible to swelling and solvation. Tables S4 and S5 indicate that for all solvents tested, the ***N*** in highly crystalline PHB and PHB-*co*-HHx (7% HHx) is much lower than that in PHB-*co*-HHx (13% HHx) and PHB-*co*-HHx (18% HHx). Additionally, chloroform and dichloromethane, which are known to be good solvents for PHAs, fail to solvate or significantly swell PHB while easily dissolving PHB-*co*-HHx (13% HHx) and PHB-*co*-HHx (18% HHx), as shown in Table S4.

**Table 1:** Molecular Weight and Thermal Properties of the Polymers Studied^a^

polymer	*M*_n_ (kDa)^a^	*M*_w_ (kDa)^a^	*D̵*^a^	*T*_g_ (°C)^c^	*T*_m_ (°C)^b^	% *X*_c_^b^	ref
PHB	283	629	2.2	3.0	169.0	58.9	
PHB-*co*-HHx (7% HHx)	442	1170	2.7	0.2	140.3	48.3	
PHB-*co*-HHx (13% HHx)	303	586	1.9	–1.1	111.8	40.3	
PHB-*co*-HHx (18% HHx)	216	403	1.9	–2.9	84.2	28.5	
PHB-*co*-4HB (>30% 4HB)	176	403	2.3	–16.1	46.7	10.8	
PLA—Ingeo 4060D^43^	117	191	1.6	59.0	N/O	0	(43)
PLA—Ingeo 4032D^37^	112	202	1.8	61.0	165.9	6.5	(37)
PLA—Ingeo 2500HP^37^	104	193	1.9	61.6	170.1	9.2	(37)
PLA—Ingeo 4950D	79	109	1.4	48.0	N/O	0	
PBS—BioPBS FZ91PM^15^	74	186	2.5	–33.7	113.4	32.5	(15)
PBSA—BioPBS FD92PM^15^	81	195	2.4	–44.8	84.8	35.2	(15)
PBAT—Ecoflex F Blend C1200^44^	26	52	2.0	–29.8	117.0	24.6	(44)
PCL—Capa 6800D^15^	120	197	1.6	n.d	60.7	56.7	(15)
PTT—Sorona bright	N/O	N/O	N/O	48.6	226.2	24.5	
PEF	N/O	N/O	N/O	71.1	209.9	3.0	

a a–Values obtained from literature or from GPC analysis, b–values measured from the melting transition of the 1^st^ heating curve of the film samples, c–values measured from the 2^nd^ heating curve, N/O—not observed, Ref.—reference.

The presence of a d-lactide isomer has similar effects in PLA. The l-lactide subunits of the polymer fold and organize into crystalline lamellae, while the majority of d-lactide subunits are excluded into the amorphous region.^40^ This phenomenon can be observed in DSC scans of higher d-lactide PLA 4060D (12% d-lactide) and PLA 4950D (45% d-lactide), which are amorphous, whereas PLA 4032D (1.5% d-lactide) and PLA 2500HP (0.5% d-lactide) are semicrystalline to varying degrees (Figure S1 and Table 1). Tables S4 and S5 reveal the effects of PLA crystallinity on the swelling and solvation of the films studied for HSP. Interestingly, PLA 4950D, a new Ingeo grade from Natureworks, LLC, that contains 45 mol % d-lactide subunits, behaves differently compared to the other PLA grades examined. Similar to 4060D, the polymer is fully amorphous, but its *T*_g_ is more than 10 °C lower at 48 °C (Figure S1 and Table 1). 4950D is also highly soluble in most of the solvents tested, except for alkanes and some alcohols.

As previously mentioned, crystallinity is not the only driving factor for polymer dissolution. Effects of molecular weight on solvation and swellability are seen in polyesters with moderate molecular weight such as PBAT, PBS, PBSA, and PCL due to their higher ***N*** values in most of the solvents used (Tables 1, S4, and S5). On the other hand, high-molecular-weight PHAs are resistant to most solvents tested (Tables 1, S4, and S5). Additionally, semiaromatic polyesters such as PEF and PTT are intrinsically more resistant to solvents compared to aliphatic polyesters.^41,42^ For these reasons, the criteria for assigning scores are determined relative to the inherent swelling behavior of each polymer, and the scoring system is subjective. However, by normalizing the solvent uptake of the polymer, the arbitrary nature of solubility studies can be limited.

### Solubility Parameters of Polyesters

Solubility parameters of polymers are typically determined by conducting a solubility study. Solvents are classified by their ability to solubilize the polymer of interest, where “good” solvents are scored 1 and “bad” solvents are scored 0. Once enough data points are collected, the software constructs a sphere in a 3-dimensional space with each axis corresponding to one of the three parameters. The sphere is placed to maximize the number of good solvents enclosed in the sphere while minimizing the number of bad solvents included. A perfect fit of 1 indicates that the sphere successfully captured all good solvents while excluding the bad solvents. The coordinates corresponding to the center of the sphere are then taken as the solubility parameters for the polymer being tested.

In many cases, a solvent may not clearly be categorized as a “good” or “bad” solvent. A solvent may swell a polymer but fail to dissolve it completely, or a polymer can become stably dispersed within the solvent, leaving a hazy solution of only a partially dissolved polymer. To accommodate these intermediate cases of solubility, the HSPiP software allows a scoring system of 1–6 to be used. Once all solvents are scored, a threshold value is chosen to determine which scores are categorized as good and which are considered bad.

The threshold can be manipulated by the user to observe its effect on the fit of the data. For example, including scores 1–4 as good solvents may result in several solvents scored 5 or 6 being included within the sphere, reducing the quality of the sphere’s fit. The user might then decide to include only solvents that scored 1–3 and find that the fit is improved by excluding those solvents that scored 4. While this method of scoring still results in a 0–1 scoring system, it allows the user to fine-tune the categorization of solvents and limit the subjectivity in their classification. Using this approach, experimentally determined solubility parameters of the polyesters are listed in Table 2, along with the radius of interaction (*R*_o_), “inside” value, and the fit of each HSP sphere.

**Table 2:** Experimentally Determined HSP of the Polyesters Studied along with the Radius of Interaction (*R*_o_), Inside Value for the Solvents Used for Calculation, and Fit of the Data to the HSP Sphere

polymer	δ_D_	δ_P_	δ_H_	δ	*R*_O_	inside	fit
PHB (0% HHx)	17.9	8.1	6.7	20.7	11.5	5	0.958
PHB-*co*-HHx (7% HHx)	17.4	8.6	6.8	20.6	10.4	5	0.960
PHB-*co*-HHx (13% HHx)	17.2	8.6	5.7	20.0	10.9	5	1.000
PHB-*co*-HHx (18% HHx)	17.3	6.4	7.0	19.7	8.6	5	0.960
PHB-*co*-4HB (>30% 4HB)	17.7	9.3	4.7	20.6	10.1	5	0.960
PLA—Ingeo 4060D	16.9	8.7	6.1	20.0	10.1	5	0.923
PLA—Ingeo 4032D	17.9	9.0	5.9	20.9	10.7	5	1.000
PLA—Ingeo 2500HP	17.7	9.2	5.9	20.8	10.2	5	0.958
PLA—Ingeo 4950D	17.6	12.5	4.8	21.9	11.4	3	0.967
PBS—BioPBS FZ91PM	17.6	8.8	6.5	20.7	11.5	4	1.000
PBSA—BioPBS FD92PM	20.9	8.1	6.3	23.3	8.5	4	1.000
PBAT—Ecoflex F Blend C1200	19.4	5.4	9.1	22.1	9.5	5	0.958
PCL—Capa 6800D	17.6	8.4	7.9	21.0	11.4	4	0.966
PTT—Sorona Bright	18.5	10.0	4.3	21.5	10.5	4	0.926
PEF	16.6	9.7	7.7	20.7	8.9	5	0.926

### Plasticizer Compatibility Study

Determining plasticizer compatibility with a polymer is one of the classic applications of HSP. To verify the quality of HSP values generated, a novel lignin-based plasticizer was synthesized with HSP values “compatible” with most of the polyesters studied (Table S6). The molecule, EP (Figure S2a), was designed using the Y-MB group contribution method in HSPiP. An “incompatible” plasticizer, DA (Figure S2b), was also identified using the plasticizer database in HSPiP. PHB-*co*-HHx (7% HHx) and PLA (Ingeo 4032D) were selected as model matrices for blending both plasticizers as plasticizers are commonly used to improve the brittleness of these polymers. Additionally, these polyesters have inherently slow crystallization kinetics without the presence of nucleating agents, which aid in generating large spherulites.^20,30,33,40^

The compatibility of these plasticizers was evaluated by calculating the plasticizer’s solubility distance (*R*_a_) from the polymers’ HSP values (Table S6). The ratio *R*_a_/*R*_o_ is known as the relative energy difference (RED) value, which indicates the affinity between a polymer and an additive. RED values closer to 0 indicate high compatibility, while those approaching 1 indicate poor compatibility.^1,34,45^ Similar to the ***N*** values of different solvents, polymers, and plasticizers, the RED values are compatible to different degrees. Less compatible plasticizers with high RED (>0.7) can be incorporated into the polymer matrix at low concentrations, but at higher concentrations, these materials may leach out or phase separate into plasticizer-rich domains.^45,46^ To understand the extent of plasticization with both molecules, the polyesters are loaded with varying amounts of plasticizer. The HSP values of EP, DA, and their compatibility with both polyesters in terms of the RED are listed in Table 3.

**Table 3:** HSP Values and Compatibility (Reported as RED) of EP and DA Plasticizers with PHB-*co*-HHx (7% HHx) and PLA (Ingeo 4032D)

material	δ_D_	δ_P_	δ_H_	δ	RED of EP	RED of DA
EP	17.4	5.2	5.7	19.0		
DA	16.2	2.4	3.4	16.7		
PHB-*co*-HHx (7% HHx)	17.4	8.6	6.8	20.4	0.34	0.72
PLA—Ingeo 4032D	16.7	8.7	4.6	20.7	0.37	0.73

**Figure 1:**
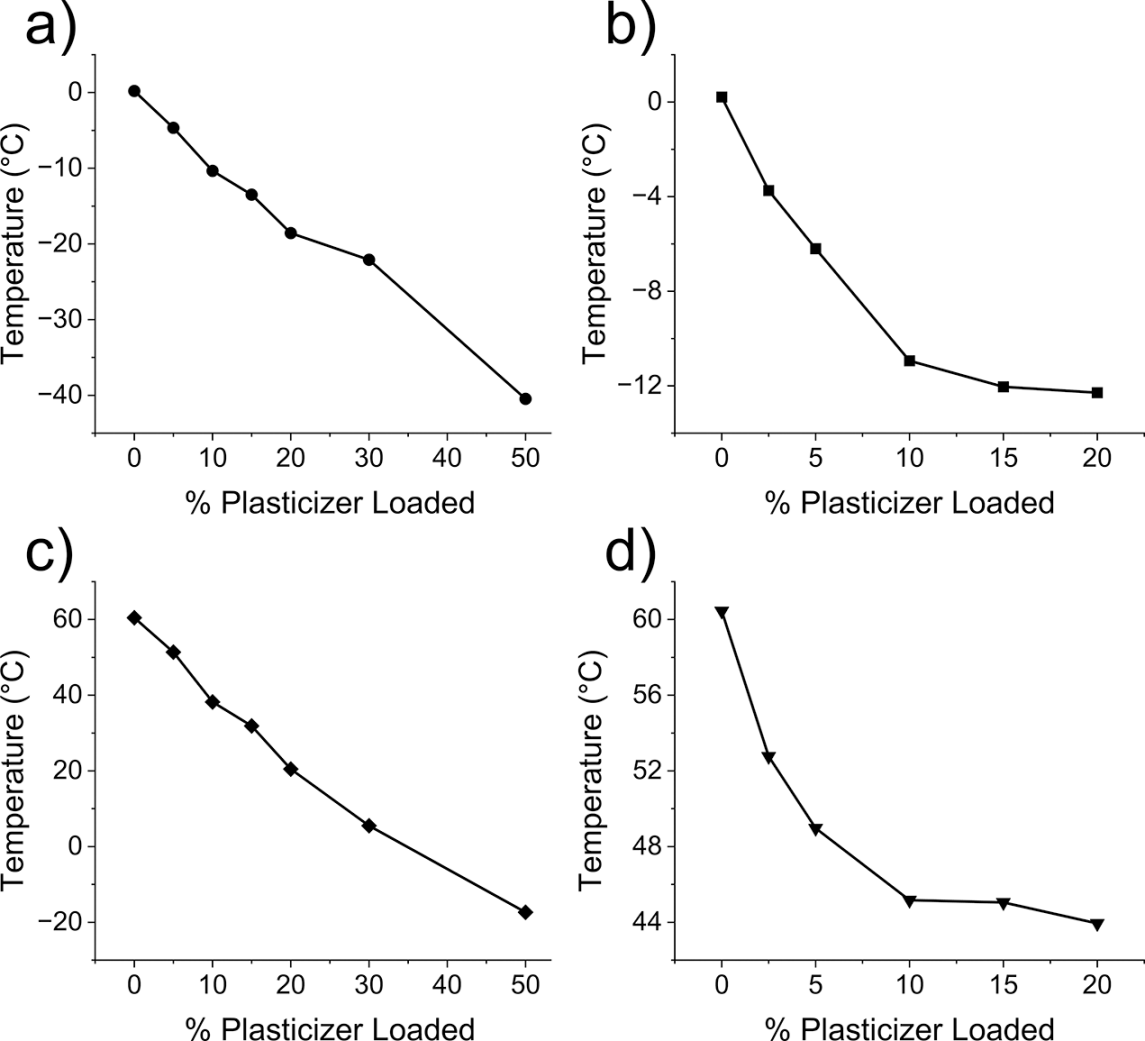
Changes in the glass transition temperature of polyesters plasticized with EP and DA at different loadings. (a) PHB-*co*-HHx (7% HHx) with EP. (b) PHB-*co*-HHx (7% HHx) with DA. (c) PLA—Ingeo 4032D with EP. (d) PLA—Ingeo 4032D with DA.

Plasticization of a polymer and plasticizer compatibility are typically observed by a shift in the glass transition temperature (*T*_g_) of the polymer. The data in Figures 1 and S2 demonstrate that both EP and DA resulted in *T*_g_ shifts in PHA and PLA, which suggests that both plasticizers are compatible with the polymers to a certain extent. For DA-loaded samples, the DSC data reveal that the extent of solubility is around 10% loading in both polyesters as the *T*_g_ depression is negligible at higher loadings. Samples loaded with EP show *T*_g_ depression all the way up to 50% concentration, resulting in a *T*_g_ of −40.5 °C in PHA and −17.4 °C in PLA (Figures 1 and S3, Table S7).

The reduction in *T*_g_ is not the sole indicator for plasticizer compatibility, especially at higher concentrations.^1,45,46^ Leaching studies are commonly conducted to further assess plasticizer compatibility, but these studies typically require large sample quantities and a specific apparatus for each standard and are time-consuming. As an alternative, POM was used to identify if phase separation was present as it is well-known that incompatible plasticizers phase separate from the polymer.^46−48^ To observe this, POM images of polymers with various loadings of EP and DA were recorded to investigate the spherulite morphology.

Large spherulites are necessary to produce optimal images for observing phase-separated domains. Spherulite size can vary significantly depending on the kinetics and thermodynamics of nucleation at a given temperature. Additives such as plasticizers also significantly impact nucleation temperatures and nucleation rates,^33,47^ as observed from the cold crystallization of plasticized samples in Figure S3. To achieve large spherulites, samples were annealed at varying times and temperatures, which were determined empirically (Table S8).

POM images of the control polymer samples and blends with a plasticizer are shown in Figures 2 and S3–S26. As predicted by HSP (Table 3), DA is incompatible with both PHB-*co*-HHx (7% HHx) and PLA (Ingeo 4032D) as it forms phase-separated domains at 2.5 and 5% loadings (Figures S5 and S18) in each polymer, respectively. Alternatively, spherulites of samples containing EP and unplasticized polymer are clear for both polyesters without any plasticizer-rich domains (Figures S4, S10–S14, S16, and S22–S25).

**Figure 2:**
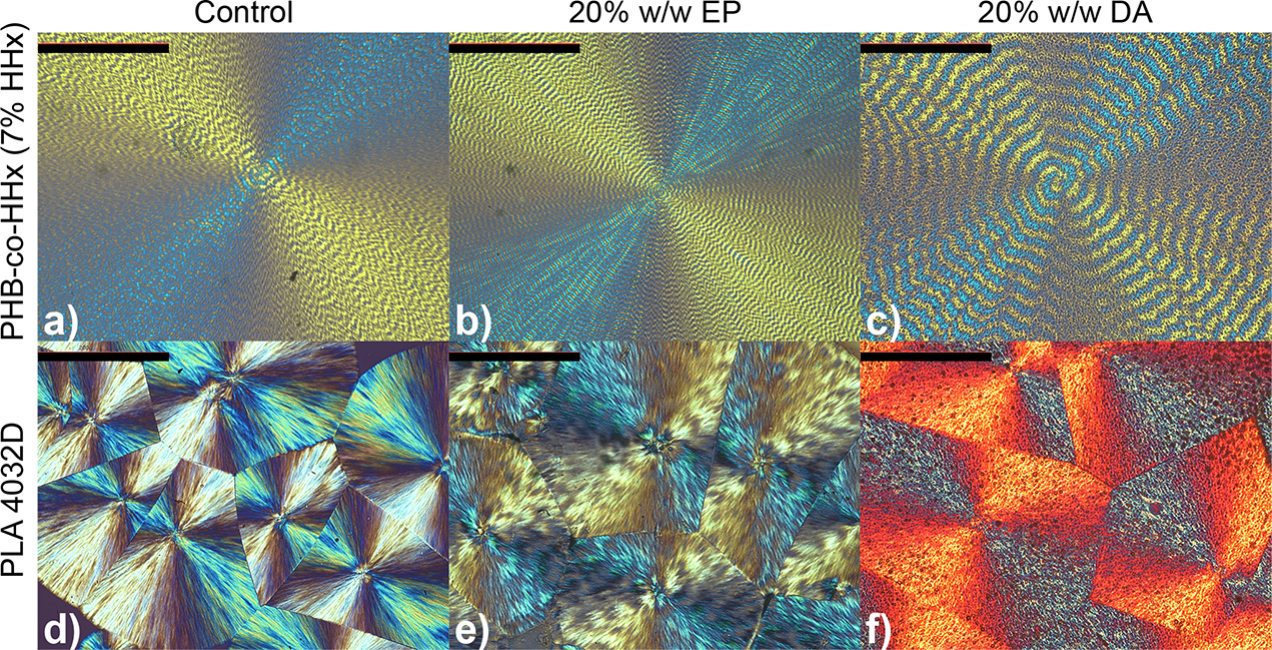
POM images of the polymer samples studied for plasticizer compatibility study (scale −200 μm). (a) PHB-*co*-HHx (7% HHx) neat. (b) PHB-*co*-HHx (7% HHx) loaded with 20% EP. (c) PHB-*co*-HHx (7% HHx) loaded with 20% DA. (d) PLA (Ingeo 4032D) neat. (e) PLA (Ingeo 4032D) loaded with 20% EP. (f) PLA (Ingeo 4032D) loaded with 20% DA.

Phase separation of DA provides further insight into the limited *T*_g_ depression from Figure 1b,d. Even though phase separation is observed at low DA concentrations, significant *T*_g_ depression is observed at loadings of 2.5% (*T*_g_ = −3.7 °C in PHA-DA-2.5 and *T*_g_ = 52.8 °C PLA-DA-2.5) and 5% (*T*_g_ = −6.2 °C in PHA-DA-5 and *T*_g_ = 49.0 °C PLA-DA-5) (Table S7). At the same concentrations, DA-5-containing samples have a lower *T*_g_ than EP-5 samples (*T*_g_ = −4.7 °C in PHA-DA-5 and *T*_g_ = 51.4 °C in PLA-DA-5). However, in samples with greater than 10% plasticizer loading, the *T*_g_ of DA remains nearly constant in both polymers, while EP continues to plasticize up to the highest loadings tested (50%, Figure 1a,c). Plasticizer-rich phases are also observed in high EP-loaded samples PHA-EP-50, PLA-EP-30, and PLA-EP-50, indicating the solubility limit in both polymers (Figures S15, S26, and S27).

## Conclusions

HSP are a powerful tool that can expedite the formulation and development of novel biobased and biodegradable polymers. However, experimental determination of HSP values for polymers is limited in accuracy due to issues with quantifying the extent of swelling. To quantify the degree of solvation, a solubility study on polymer film samples was conducted with common organic solvents. Using eq 1, normalized solvent uptake (***N***) was calculated as a function of the mass difference between swollen and dry films and density. Solvent compatibility determined from ***N*** in conjunction with algorithms available in HSPiP software was used to determine accurate HSP values for each polyester.

The predictive powers of HSP and HSPiP were highlighted by designing a novel lignin-based plasticizer compatible with all of the polyesters studied. Theoretical compatibility of EP, determined from the Y-MB group contribution, was experimentally verified using DSC and POM with PHB-*co*-HHx and PLA. An incompatible plasticizer, DA, was also predicted from the HSPiP database to show the effects of less compatible plasticizers in polymers. The incompatible nature of DA limited its incorporation into the polymer matrix, as observed by POM, and resulted in a loss of *T*_g_ depression at higher loadings in both polymers. In contrast, EP did not phase-separate and continued to depress *T*_g_ up to the highest loading tested. Further research into polymer HSP and theoretical HSP of small molecules can expand the potential applications of biopolymers in several fields as well as expedite the development of novel replacements of toxic additives used in the plastics industry.

## Supporting Information Available

Definitions and standards for degradation testing, synthetic procedures for small-molecule plasticizers and PEF, ^1^H NMR of plasticizers, DSC thermograms of the polyesters studied, heat of fusion for % crystallinity calculations, HSP scoring criteria for each polymer and scores assigned to each solvent studied, HSP compatibility of both plasticizers with bioplastics, isothermal crystallization temperatures for POM images, and POM images of PHB-*co*-HHx (7% HHx) and PLA (Ingeo 4032D) loaded with plasticizers (PDF)
