# Supplementary material for: Experimentally Determined Hansen Solubility Parameters of Biobased and Biodegradable Polyesters
Source: ACS Sustain Chem Eng. 2024 Feb 1;12(6):2386–93. doi: 10.1021/acssuschemeng.3c07284 (PMC10865435; doi:10.1021/acssuschemeng.3c07284)
Supplement: Supplementary file 1 — sc3c07284_si_001.pdf [file sc3c07284_si_001.pdf]

Supporting Information for:

Experimentally Determined Hansen Solubility Parameters of Biobased and Biodegradable Polyesters

Kush G. Patel[†,§], Ryan K. Maynard[‡,§], Lawrence S. Ferguson IV[§], Michael L. Broich II[†,§], Joshua C. Bledsoe[‡,§], Caitlin C. Wood[‡,§], Grant H. Crane[§], Jessica A. Bramhall[§], Jonathan M. Rust[§], Amanda Williams-Rhaesa[§], and Jason J. Locklin[†,‡,§,*]

† School of Chemical, Materials, and Biomedical Engineering, College of Engineering, University of Georgia, *Athens, GA 30602.*

‡ Department of Chemistry, Franklin College of Arts and Sciences, University of Georgia, *Athens, GA, 30602*.

§ New Materials Institute, University of Georgia, *Athens, GA, 30602.*

* Corresponding Author – email: jlocklin@uga.edu

Number of pages: 38

Number of tables: 8

Number of figures: 29

Table of Contents

Biodegradation Standards and Methods — Table S1

Biodegradability of polyesters studied — Table S2

Heat of fusion (ΔH_0) values of polyesters studied — Table S3

HSP scoring criteria for each polymer — Table S4

HSP scores assigned for each polymer — Table S5

HSP distances and RED of both plasticizers with all polyesters — Table S6

Data from DSC Scans of plasticized polyesters — Table S7

Isothermal crystallization times of plasticized and unplasticized polyester samples — Table S8

DSC thermograms of all polyesters — Figure S1

Chemical structures of plasticizers — Figure S2

DSC thermograms of plasticized polyesters — Figure S3

POM Image of control PHB-*co*-HHx (7% HHx) — Figure S4

POM Images of PHB-*co*-HHx (7% HHx) with DA — Figures S5-S9

POM Images of PHB-*co*-HHx (7% HHx) with EP — Figures S10-S15

POM Image of control PLA (Inego™ 4032D) — Figure S16

POM Images of PLA (Inego™ 4032D) with DA — Figures S17-S21

POM Images of PLA (Inego™ 4032D) with EP — Figures S22-S27

^{1}H-NMR Spectra of EHP — Figure S28

^{1}H-NMR Spectra of EP — Figure S29

Definitions and Standards for Biodegradation of Polymers

Degradability of plastic products is a property desirable for many single-use articles which accumulate and contaminate the environment. However, the mechanisms for plastic degradation depend on numerous factors including oxidation, UV degradation, and microbial degradation. Many review articles have been published providing detailed information on several types and mechanisms of polymer degradation. For background information on the topic of polymer biodegradation, we have included definitions that the New Materials Institute has adopted, including the trademarked term *Bioseniatic™* first introduced in the lexicon in 2019.

Degradable plastic: A material that will undergo a substantial change in chemical structure under certain specific environmental conditions, resulting in a change in the material properties such as fragmentation, thermomechanical properties, and/or discoloration. Degradable plastics are not necessarily biodegradable or compostable. The process is better described as micronization.

Biodegradable: a degradable plastic in which the degradation results from the action of naturally occurring microorganisms such as bacteria, fungi, and algae. The process of biodegradation depends on the surrounding environment (moisture, temperature, inoculum, microbial load) and on the material itself.

Compostable: a plastic that undergoes degradation by biological processes during composting to yield CO2, water, inorganic compounds, and biomass at a rate consistent with other known compostable materials and leaves no visible, distinguishable, or toxic residue.

Bioseniatic™: a naturally-sourced or synthetically-derived polymer with no additives or chemical modifications to their structure that prevent them from being biologically converted into a non-polymeric form of naturally occurring, non-toxic compounds at a rate congruous with natural analogues.

In addition to these definitions, several national and international organizations have developed testing methods and standards for defining composability and biodegradation of polymers. Among these organizations, TÜV AUSTRIA has defined criteria which are used as basis by ASTM, ISO, DIN, EN, AS, and others as described.[1, 2] Detailed information on specimen type, environmental conditions, and test period for ASTM and ISO standards are listed in Table S.1. Additionally, the polymers studied are also classified by their status measured by the standards in Table S.2.

Table S1. Biodegradation conditions, sample properties and pass/fail criteria described by standardized testing methods from ASTM and ISO. (‘+’ indicates positive control and ‘-’ indicates negative control used for these studies.)

Standard	Environment	Sample size	Sample geometry	Controls	Temperature, °C	pH	Test time
**ASTM D6691**	Aerobic, Marine	20 ± 0.1 mg	Powder, Film, Fragments, Formed articles,	+: cellulose, chitin, or kraft paper<br>-: solitary inoculum	30 ± 2	n/a	10-180 days

			Aqueous solution				
**ASTM D5271**, **ISO 14851**	Aerobic, Activated wastewater treatment sludge	at least 60 mg/mL	Films, Fragments, Formed articles	+: analytical-grade cellulose for TLC<br>-: polyethylene	23 ± 2	7.0± 0.2	1-6 months
**ASTM D5511, ISO 15985**	Anaerobic, High-solids anaerobic digestion	up to 100g dry weight basis	Film, Powder, Pellet, Formed article, Dog bone	+: analytical-grade cellulose for TLC<br>-: polyethylene	52 ± 2 (thermophilic)<br>37 ± 2 (mesophilic)	7.5-8.5	15-30 days
**ASTM D5338, ISO 14855**	Aerobic, Controlled composting	2 x 2 cm max	Film, Powder, Granule, Formed article, Dog bone	+: analytical-grade cellulose for TLC<br>-: polyethylene	58 ± 2	7-8.2	45 days
**ASTM D5988, ISO 17556**	Aerobic, Soil	enough to provide 200-1000 mg carbon for 500g soil	Film, Fragment, Powder, Formed article, Aqueous solution	+/-: required, but not specified	20-28 ± 2	6.0-8.0	until no net CO_2 production is noted between measurements 4 weeks apart

Table S2. Classification of polymers studied by biodegradability in various conditions described by standards.

**Standard(s)**	**Polymers**
**ASTM D5338, ISO 14855**	PLAs, PBS, PBSA, PBAT, PHAs, and PCL
**ASTM D5511, ISO 15985**	PLAs, PHAs
**ASTM D5988, ISO 17556**	PHAs, PCL, PBSA, PBAT (certain grades)
**ASTM D5271, ISO 14851**	PHAs
**ASTM D6691**	PHAs

Supplementary Data and Results

Synthesis

Synthesis of dimethyl-2,5-dicarboxylate:

A solution containing 40.0 g of 2,5-furandicarboxylic acid, 10.0 mL of concentrated H_2SO_4, and 1.0 L of methanol was refluxed for 16 hours in air. The solution was precipitated into cold water, and the resulting solid was filtered, washed with saturated sodium bicarbonate, rinsed with water, then dried under reduced pressure at 60°C for 24 hours. 36.6 g of an off-white powder was isolated (78%). ^1H NMR (600 MHz, $CDCl_3$) δ 7.22 (s, 2H), 3.93 (s, 6H).

Synthesis of poly(ethylene furanoate) (PEF):

14.5 g of 2,5-dimethylfurandicarboxylate (1.0 eq.) and 14.67 g of ethylene glycol (3.0 eq.) were added to a 100 mL three-necked flask equipped with a distillation apparatus and mechanical stirrer. The flask was evacuated under full vacuum and backfilled five times with dry nitrogen before being heated to 160°C. Once the contents of the flask were homogenous, 67.0 mg of titanium tert-butoxide (0.5 mol % relative to diester) was dissolved in 2.0 mL toluene and added *via* syringe while stirring at 150 RPM. The reaction was heated to 200°C at a rate of 10°C/hr under nitrogen. Vacuum was then slowly applied over 30 minutes until pressure was sustained below 200 mTorr. The temperature ramp was increased to 20°C/hr until 240°C, at which point the viscosity limit was reached for the apparatus. The flask and contents were cooled under vacuum to room temperature, then 75 mL of hexafluoro-2-propanol were added. The resulting solution was precipitated into 1 L cold methanol. The precipitate was dried under reduced pressure for 24 hours yielding 12.7 g of an off-white polymer (89%).

Synthesis of ethyl 3-(4-hydroxy-3-methoxyphenyl)propanoate

Hydroferulic acid was synthesized as previously reported.[3] 1.94 g of hydroferulic acid (10 mmol), 150 mL of dry ethanol, and 0.1 mL of concentrated sulfuric acid were added to a 500 mL 2-neck flask under nitrogen and refluxed for 5 hours. The solvent was removed under reduced pressure and the residue was dissolved in ethyl acetate. The organic layer was washed three times with water and saturated sodium bicarbonate, then once with brine. The organic layer was dried over magnesium sulfate, filtered, and residual solvent was removed under reduced pressure to afford 1.90 g of ethyl 3-(4-hydroxy-3-methoxyphenyl)propanoate as a clear oil (85%). ^1H NMR (600 MHz, $CDCl_3$) δ 6.83 (d, 1H), 6.71 (s, 1H), 6.69 (d, 1H), 5.50 (s, 1H), 4.21 (q, 2H), 3.87 (s, 3H), 2.88 (t, 2H), 2.59 (t, 2H), 1.24 (t, 3H).

Synthesis of ethyl 3-(4-ethoxy-3-methoxyphenyl)propanoate (EP)

1.12 g of ethyl 3-(4-hydroxy-3-methoxyphenyl)propanoate (1.0 eq.), 2.76 g of anhydrous potassium carbonate (4.0 eq.), 20 mL of dry DMF, and 1.08g of bromoethane (2 .0 eq.) were added to a pressure vessel and sealed. The contents of the flask were heated while stirring at 60°C for 12 hours before being combined with 200 mL of distilled water. The aqueous mixture was extracted with ethyl acetate then washed with brine and dried over magnesium sulfate. The mixture was filtered and the solvent was evaporated under reduced pressure to yield 1.23 g of ethyl 3-(4-ethoxy-3-methoxyphenyl)propanoate as a light tan oil (96%). ^1H NMR (600 MHz, $CDCl_3$) δ 6.79 (d, 1H), 6.72 (t, 2H), 4.09 (m, 4H), 3.86 (s, 3H), 2.89 (t, 2H), 2.60 (t, 2H), 1.44 (t, 3H), 1.24 (t, 3H).

Differential Scanning Calorimetry (DSC)

Table S3. Heat of fusion (ΔH_0) values used to determine % crystallinity of polyesters.

**Polymer(s)**	**Heat of fusion (ΔH_0)**
PHB-*co*-HHx (0% to 18% HHx and 4HB)	146 J/g[4]
PLA (Ingeo™ 2500HP and 4032D)	93.7 J/g[5]
PBS (BioPBS™ FZ91PM)	200 J/g[6]
PBSA (BioPBS™ FD92PM)	110.3 J/g[7]

PBAT (Ecoflex® F Blend C1200)	114 J/g[8]
PCL (Capa® 6800D)	139.5 J/g[9]
PTT (Sorona® Bright)	145.6 J/g[10]
PEF	137 J/g[11]

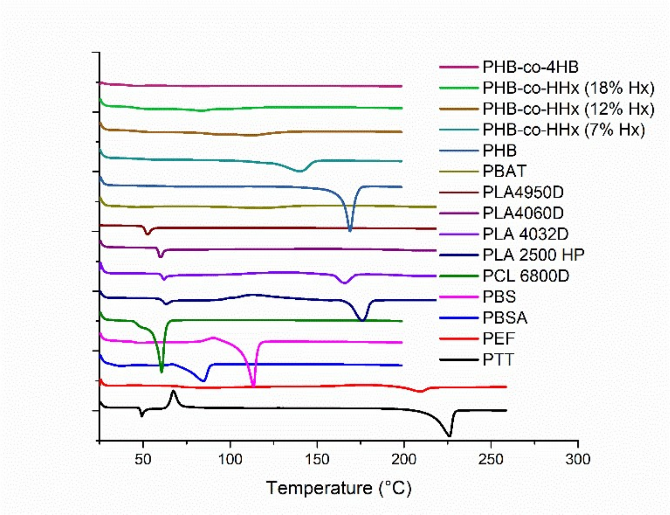

Figure S1. DSC thermograms of the first heating curve of the polymer film samples used in this study.

Solubility Study Data

Table S4. HSP scoring criteria for each polymer studied. All values listed are in normalized solvent uptake $\left(\frac{\mu L}{g}\right)$. (ImDis – Dissolution of film within 1 hour; DisD – Dissolution of film within 24 hours; DisT – Dissolution of film within the duration of testing; SwlUnrec – Film swells to such an extent that it is no longer recoverable)

Score	PHB - 0HHx	PHB - 7HHX	PHB - 12HHx	PHB - 18HHx	PHB - 30-4HB	PLA - 4950D	PLA - 4060D	PLA - 4032D	PLA – 2500HP	PBS	PBSA	PBAT	PCL	PTT	PEF
1	DisT	DisT	DisD	DisD	ImDis	ImDis	ImDis	DisT	DisT	DisD	DisD	DisD	ImDis	DisT	DisT
2	> 200	Swl Unrec	DisT	DisT	DisD	DisD	DisD	> 1000	> 700	DistT	> 1000	DisT	DisD	> 200	Swl Unrec
3	100 – 200	> 150	> 400	Swl Unrec	DisT	DisT	DisT	500 – 1000	400 – 700	> 100	500 – 1000	Swl Unrec	DisT	100 – 200	500 – 1500
4	50 – 100	75 – 150	400 – 200	200 – 2000	Swl Unrec	Swl Unrec	Swl Unrec	250 – 500	150 – 400	50 – 100	150 – 500	> 250	> 250	50 – 100	250 – 500
5	10 – 50	25 – 75	50 – 200	50 – 200	> 500	> 500	> 250	50 – 250	50 – 150	10 – 50	50 – 150	100 – 250	100 – 250	10 – 50	100 – 250
6	< 10	< 25	< 50	<50	< 500	< 500	< 250	< 50	< 50	< 10	< 50	< 100	< 100	< 10	< 100

Table S5. HSP scores assigned to solvents for each polyester according to criteria defined in Table S2.

Solvents	PHB - 0HHx	PHB - 7HHx	PHB - 13HHx	PHB - 18HHx	PHB – 30-4HB	PLA - 4950D	PLA - 4060D	PLA - 4032D	PLA – 2500HP	PBS	PBSA	PBAT	PCL	PTT	PEF
Chloroform	4	1	1	1	1	1	1	1	1	1	1	1	1	3	2
Xylene	4	4	5	4	5	4	3	2	4	4	5	5	1	5	6
Toluene	4	4	5	4	5	3	2	3	4	4	6	6	1	4	6
Cyrene™	-	-	-	-	-	-	-	-	-	-	2	4	-	-	-
Anisole	3	3	-	3	4	2	2	3	3	3	-	3	1	3	4
Acetone	-	4	4	3	4	3	2	4	4	4	6	6	3	4	5
Benzene	4	4	5	3	4	2	2	1	2	4	5	5	1	3	6
D-Limonene	5	6	5	5	6	-	6	5	6	5	5	1	5	6	4
Dichloromethane	5	4	1	1	3	1	1	1	5	1	1	4	1	4	2
Benzyl benzoate	5	-	5	4	5	-	3	3	4	4	4	4	2	5	5
N-Methyl-Pyrrolidone	4	3	2	1	4	2	2	3	3	3	2	2	2	3	3
1,3-Dioxolane	4	2	2	2	2	2	1	1	1	3	1	1	1	3	2
Butyl Benzoate	2	-	5	4	5	-	4	4	4	4	4	4	5	5	6
Tetrahydrofuran	3	3	2	2	4	2	2	3	4	4	6	1	1	3	5
Acetonitrile	5	4	3	3	4	2	2	3	4	4	6	6	4	4	5
Cyclohexanone	4	3	4	3	4	2	2	3	2	3	4	4	2	5	4
Dimethyl Sulfoxide	3	4	5	6	4	3	5	5	5	4	6	6	6	6	4
Dimethyl Formamide	4	4	4	3	4	2	3	3	4	3	5	5	5	3	3
1,2-Dichloroethane	-	-	1	-	-	-	2	-	-	3	1	2	1	-	-
Heptane	6	6	6	6	6	6	6	6	6	6	6	6	6	6	6
Methyl Ethyl Ketone	3	4	-	3	4	3	-	4	4	5	5	5	2	4	5
1,2-Dichlorobenzene	4	4	-	3	4	3	-	3	3	-	-	-	-	6	5
Ethyl Acetate	-	6	5	-	-	-	3	-	-	-	6	-	-	-	-
Dimethyl Carbonate	-	6	-	-	-	-	-	-	-	-	-	-	-	-	-
n-Octanol	-	5	-	-	-	-	-	-	-	-	-	-	-	-	-

Acetic Acid	-	-	-	-	-	2	3	3	-	3	-	-	1	-	4
Methanol	-	-	-	-	-	5	-	-	-	-	-	-	-	-	-
Ethanol	-	-	-	-	-	5	-	-	-	-	-	-	-	-	-
n-Propanol	5	5	5	-	-	6	-	-	6	-	-	-	-	-	-
n-Butanol	4	-	5	6	6	6	6	6	6	5	-	6	-	-	-
Acetic Anhydride	-	3	-	-	-	-	-	-	-	-	-	4	5	-	-
Chlorobenzene	-	-	-	-	4	-	-	-	-	-	3	-	-	-	6
Aniline	-	1	-	2	3	2	3	-	-	-	-	-	1	3	2
1,4-Dioxolane	-	-	-	-	-	2	-	-	-	-	-	-	1	3	3
Cyclohexane	5	6	6	6	-	6	6	6	6	5	-	-	6	6	-
Morpholine	-	3	-	-	-	-	-	-	-	-	-	-	1	-	-
m-Cresol	-	1	-	1	3	2	3	-	-	-	-	-	1	2	2
2-Methyl-2-Butanol	-	-	-	-	6	6	6	-	-	-	-	5	-	-	-
2-Ethyl Hexanol	-	-	-	-	-	6	6	-	-	-	-	-	5	6	5
Formic Acid	-	-	-	-	-	3	-	-	-	-	-	-	-	-	-
Propylene Carbonate	3	5	4	-	-	3	-	3	4	3	-	-	5	5	-
Butyric Acid	-	-	-	-	-	-	-	-	-	-	-	-	-	6	6

HSP and Plasticizer Compatibility with polyesters

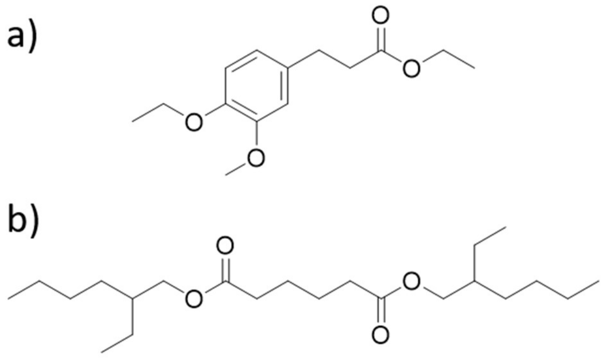

Figure S2. Plasticizers for compatibility studies. a) Ethyl 3-(4-ethoxy-3-methoxyphenyl)propanoate (EP) and b) Di-(2-ethylhexyl)adipate (DA).

Table S6. HSP distances (R_a) and RED values of EP and DA with all polyesters studied.

Polymer	EP Distance	EP RED	DA Distance	DA RED
PHB (0% HHx)	3.23	0.28	7.41	0.64
PHB-*co*-HHx (7% HHx)	3.57	0.34	7.47	0.72
PHB-*co*-HHx (13% HHx)	3.42	0.31	6.91	0.63
PHB-*co*-HHx (18% HHx)	1.78	0.21	5.81	0.68
PHB-*co*-4HB (> 30% 4HB)	4.26	0.42	7.64	0.76
PLA – Ingeo™ 4950D	7.26	0.64	10.43	0.91
PLA – Ingeo™ 4060D	3.66	0.36	7.00	0.69
PLA – Ingeo™ 4032D	3.92	0.37	7.83	0.73
PLA – Ingeo™ 2500HP	4.05	0.40	7.84	0.77
PBS – BioPBS™ FZ91PM	3.71	0.32	7.64	0.66
PBSA – BioPBS™ FD92PM	4.72	0.56	8.62	1.01
PBAT – Ecoflex® F Blend C1200	4.93	0.52	9.18	0.97
PCL – Capa® 6800D	3.90	0.34	8.01	0.70
PTT – Sorona® Bright	5.46	0.52	8.93	0.85
PEF	5.18	0.58	8.51	0.96

DSC Thermograms of Unplasticized and Plasticized Polarized Optical Microscopy Samples

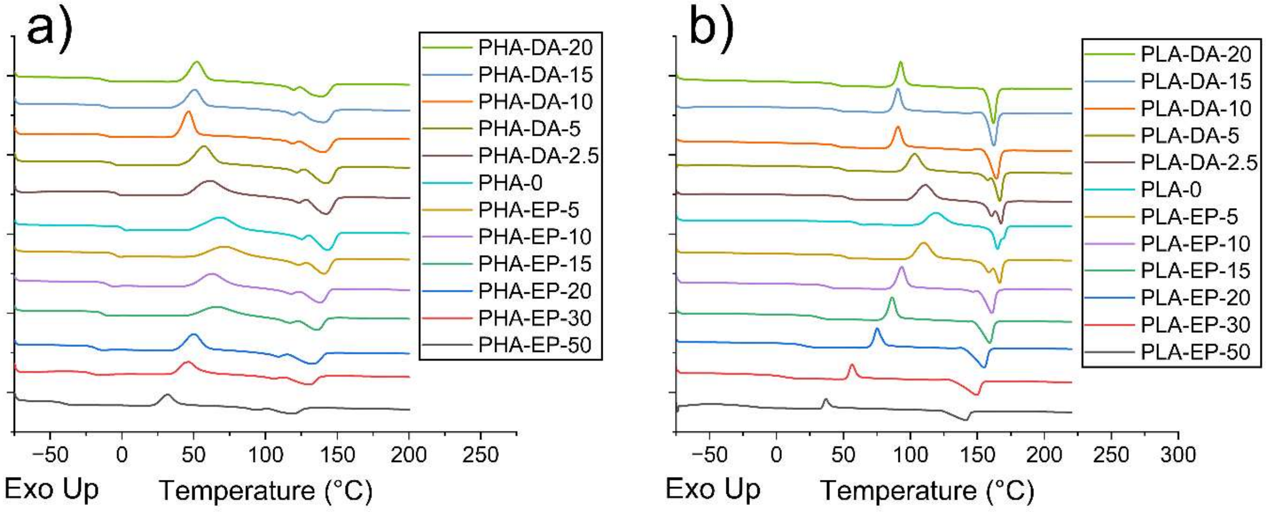

Figure S3. Non-isothermal DSC Thermograms of film samples of neat and plasticized (a) PHB-*co*-HHx (7% HHx) and (b) PLA – Ingeo™ 4032D. (PHA-0 indicates unplasticized PHB-*co*-HHx (7% HHx); PLA-0 indicates unplasticized PLA – Ingeo™ 4032D; PHA-EP-x samples are plasticized with EP and x indicates the % loading of plasticizer; PHA-DA-x samples are plasticized with DA and x indicates the % loading of plasticizer; PLA-EP-x samples are plasticized with EP and x indicates the % loading of plasticizer; PLA-DA-x samples are plasticized with DA and x indicates the % loading of plasticizer.)

Table S7. Data from non-isothermal DSC Scans of PHB-*co*-HHx (7% HHx) and PLA – Ingeo™ 4032D with samples plasticized using EP and DA. T_m and % crystallinity (% X_c) were obtained from 1st heating curve. Glass transition temperature (T_g) and (T_{cc}) were obtained from the 2nd heating curve.

**Sample**	**T_g**	**T_m**	**% X_c**	**T_{cc}**
PLA-0	60.5	162.4	29.4	119.9
PLA-EP-5	51.4	165.4	43.5	110.2
PLA-EP-10	38.2	161.3	39.3	93.6
PLA-EP-15	31.9	159.4	40.5	86.4
PLA-EP-20	20.5	154.5	39.5	70.8
PLA-EP-30	5.5	149.4	44.8	56.4
PLA-EP-50	-17.4	141.0	44.4	36.9
PLA-DA-2.5	52.8	166.8	30.7	111.6
PLA-DA-5	49.0	166.2	30.0	103.3
PLA-DA-10	45.2	165.3	30.2	90.8
PLA-DA-15	45.1	163.3	33.9	90.8
PLA-DA-20	43.9	162.5	31.2	92.7
PHA-0	0.2	142.4	46.7	69.3
PHA-EP-5	-4.7	139.7	49.9	72.5
PHA-EP-10	-10.4	137.6	49.7	63.5
PHA-EP-15	-13.5	134.9	48.9	67.0
PHA-EP-20	-18.6	133.1	48.1	50.4
PHA-EP-30	-22.1	129.3	43.3	46.3
PHA-EP-50	-40.5	119.0	47.6	32.2
PHA-DA-2.5	-3.7	140.3	42.8	61.9
PHA-DA-5	-6.2	141.1	49.4	57.6
PHA-DA-10	-10.9	138.3	42.3	46.4
PHA-DA-15	-12.0	138.5	49.7	50.7
PHA-DA-20	-12.3	138.7	44.7	52.3

Table S8. Isothermal crystallization times (t_C) and temperatures (T_C) of control and plasticized PHB-*co*-HHx (7% HHx) and PLA (Ingeo™ 4032D) used for POM.

**Sample**	**PHB-*co*-HHx (7% HHx)**		**PLA (Ingeo™ 4032D)**	
	**t_C (min)**	**T_C (˚C)**	**t_C (min)**	**T_C (˚C)**
Control	60	95	60	130
5% EP	60	80	60	120
10% EP	60	80	60	120
15% EP	60	80	60	120
20% EP	60	85	60	120
30% EP	60	70	60	100
50% EP	60	70	60	100
2.5% DA	60	80	60	120
5% DA	60	80	60	120
10% DA	60	80	60	120
15% DA	60	80	60	120
20% DA	60	90	60	120

Full Size Polarized Optical Microscopy Images

The POM images displayed in Figure 2 are fitted to accommodate the author guidelines of the publication. Due to the smaller size of the images, viewing the phase separation observed in DA loaded sample is difficult. The phase separated domains of DA are dispersed throughout the spherulite surface with increasing concentrations. Phase separated domains of EP at higher plasticizer loadings are much smaller than DA domains with only small amounts of separation. Thus, full size images of the control, EP loaded, and DA loaded polymers spherulites are included in the following Figures S4-S27.

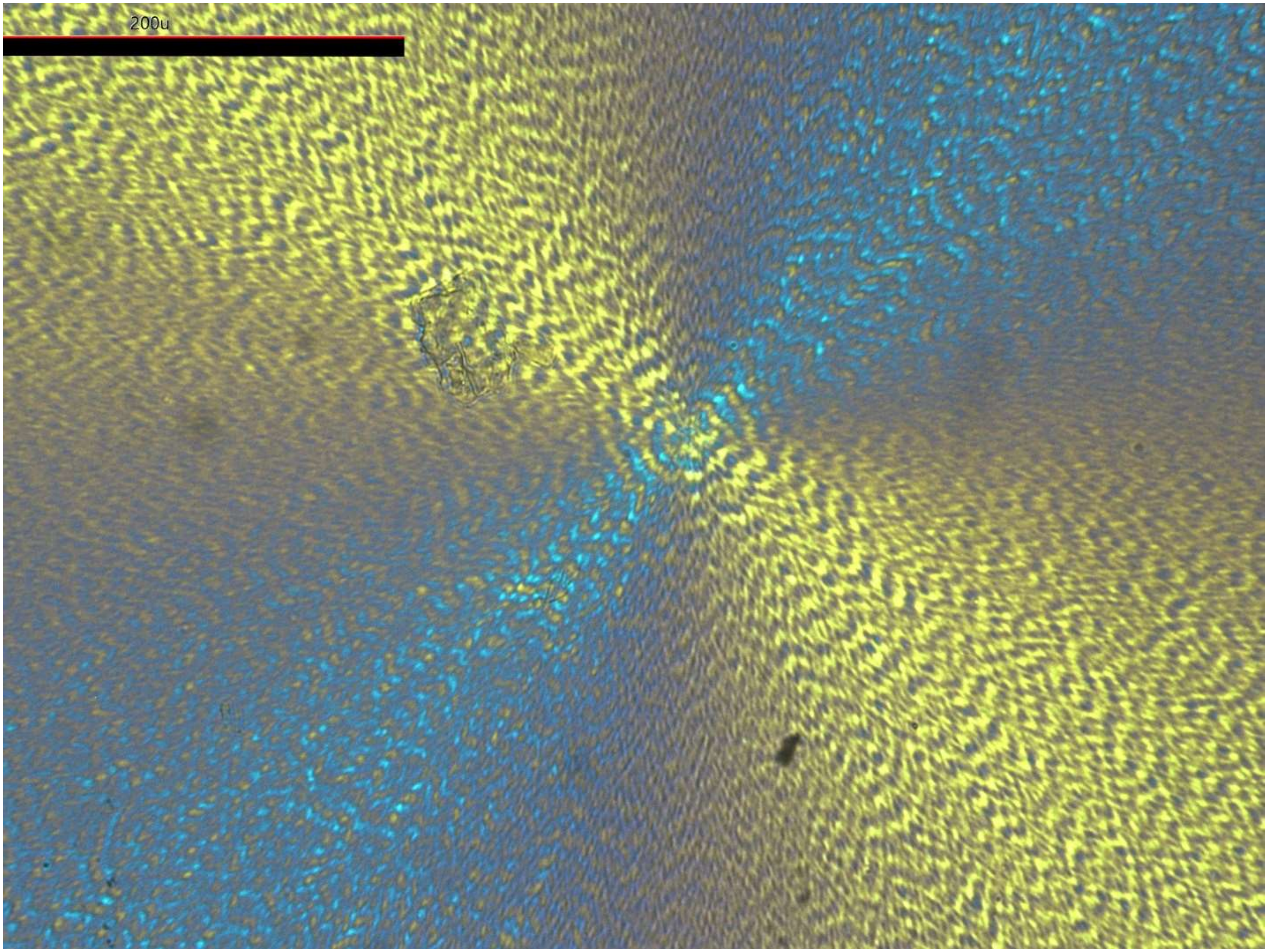

Figure S4. POM Image of PHB-*co*-HHx (7% HHx) control spherulite (scale – 200 µm).

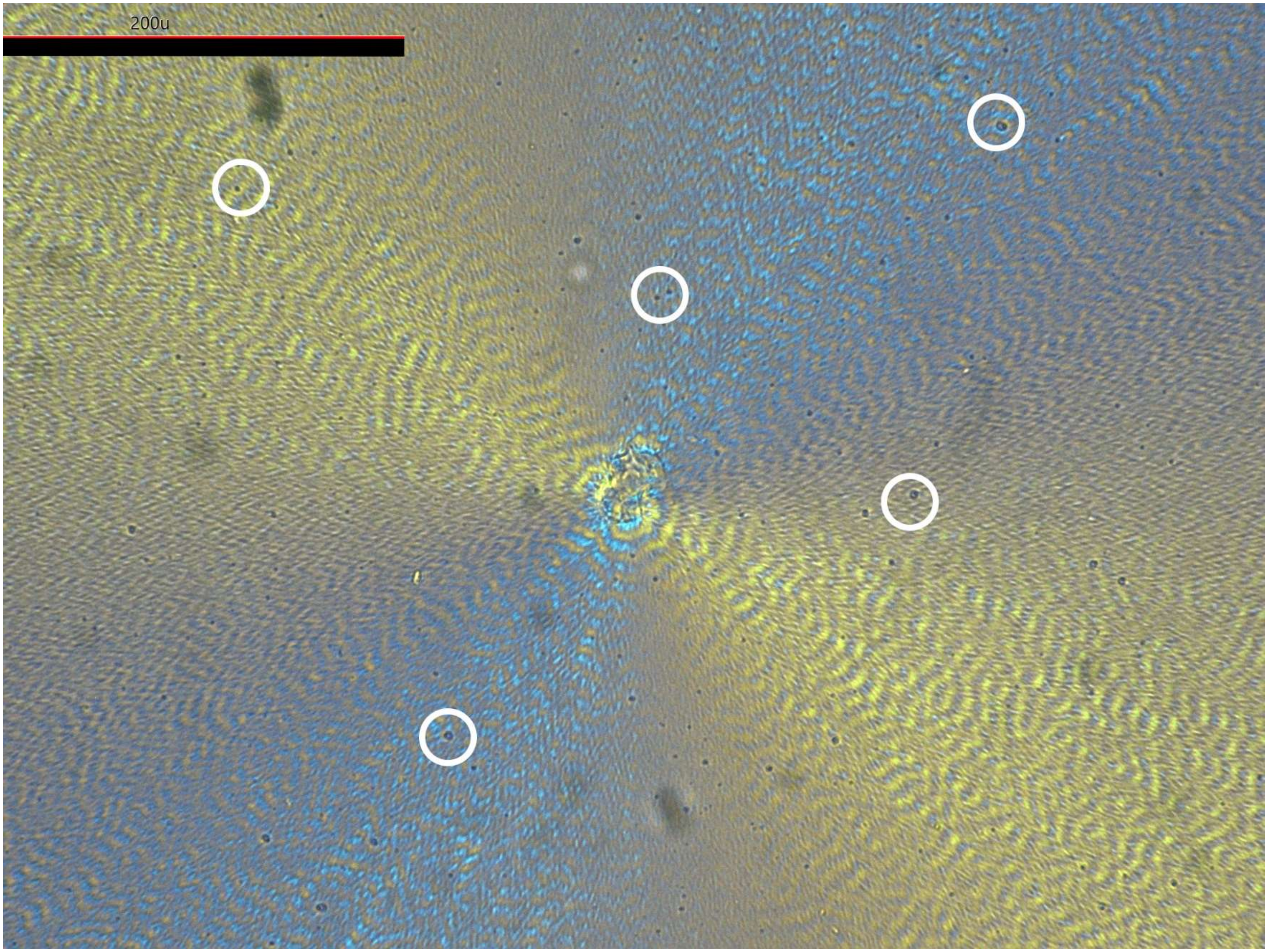

Figure S5. POM Image of PHB-*co*-HHx (7% HHx) loaded with 2.5% w/w DA spherulite (scale – 200 µm). White circles indicate some of the phase separated domains which appear as grainy texture on the spherulite surface.

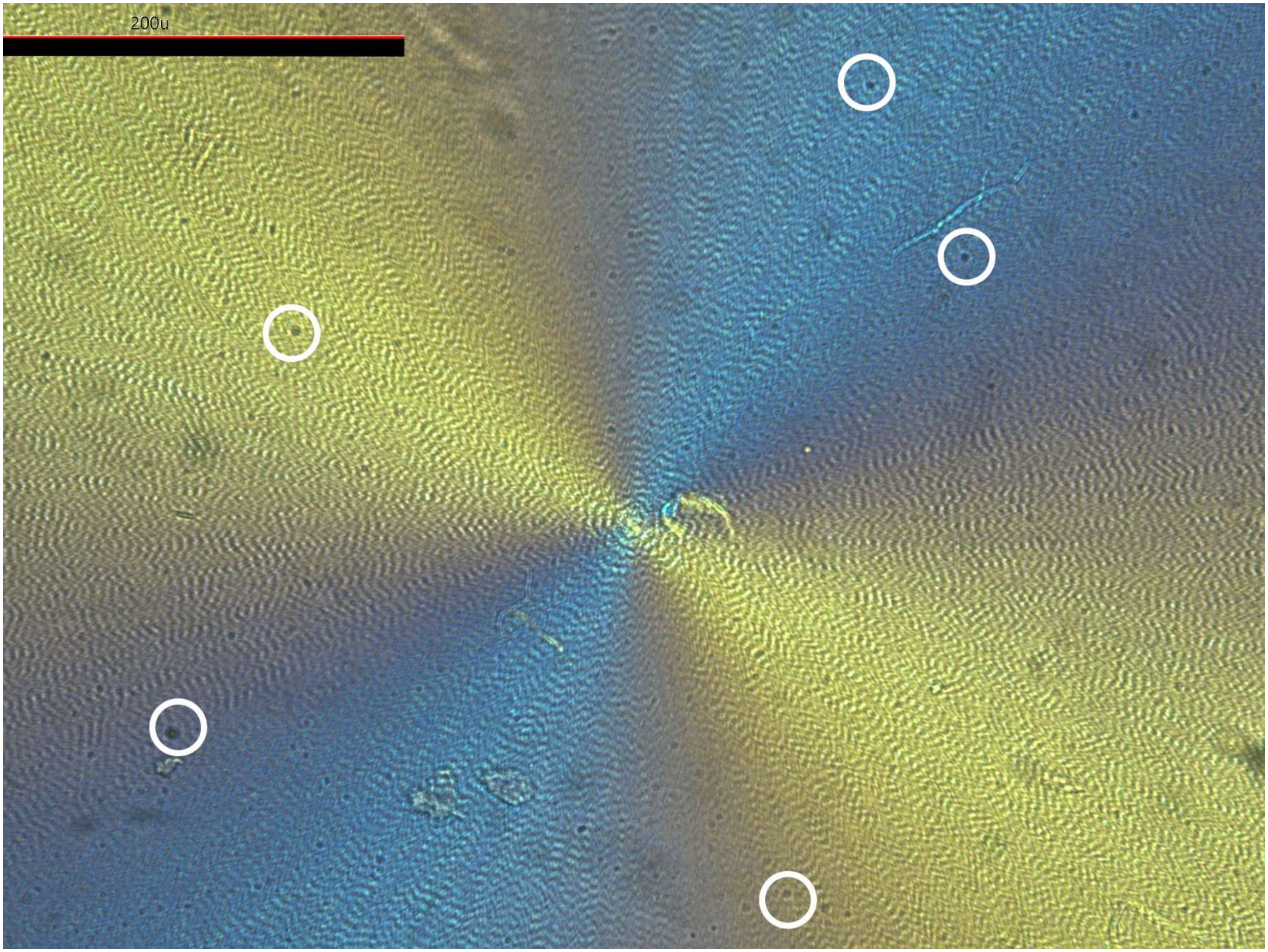

Figure S6. POM Image of PHB-*co*-HHx (7% HHx) loaded with 5% w/w DA spherulite (scale – 200 µm). White circles indicate some of the phase separated domains which appear as grainy texture on the spherulite surface.

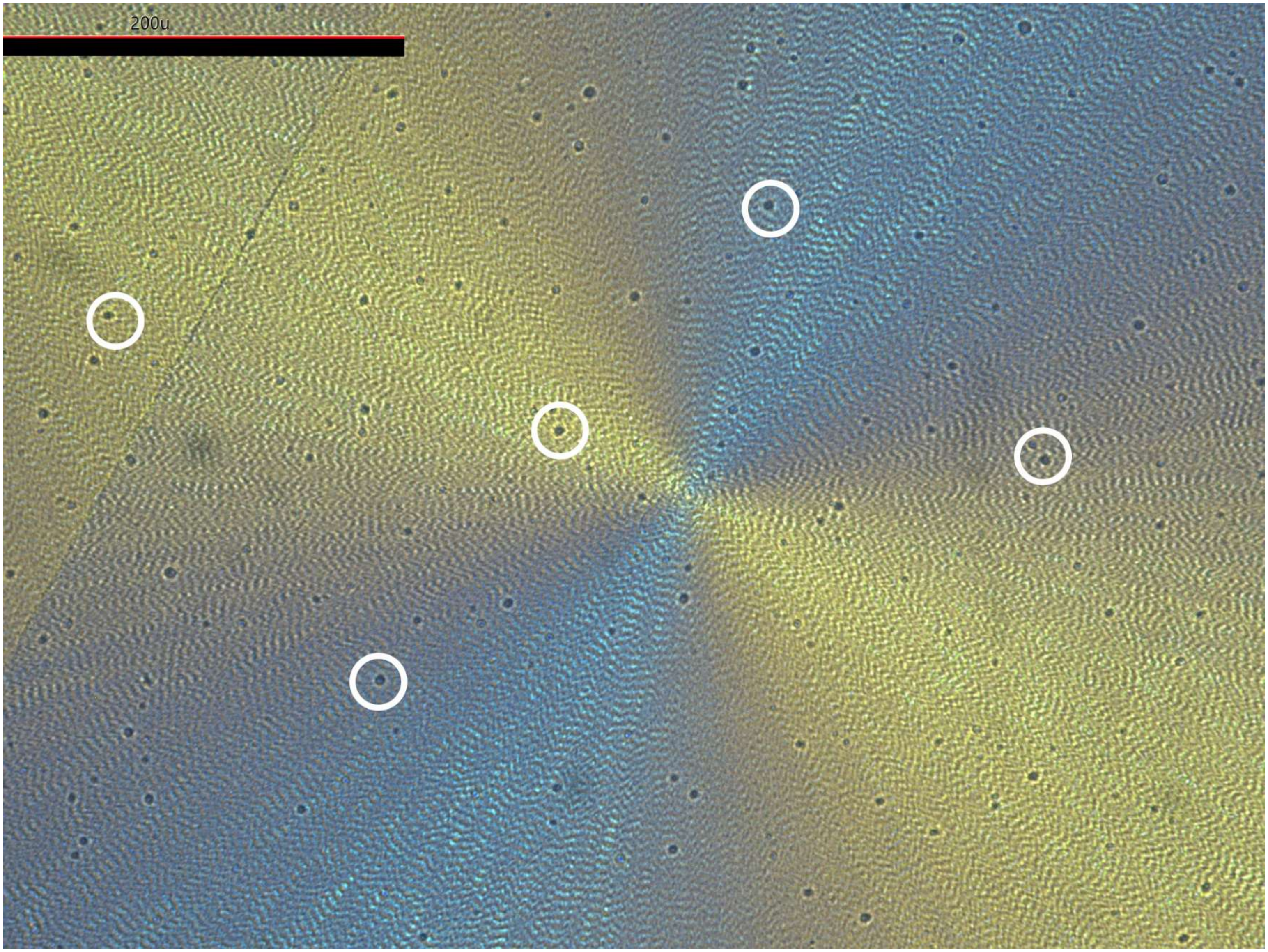

Figure S7. POM Image of PHB-*co*-HHx (7% HHx) loaded with 10% w/w DA spherulite (scale – 200 µm). White circles indicate some of the phase separated domains.

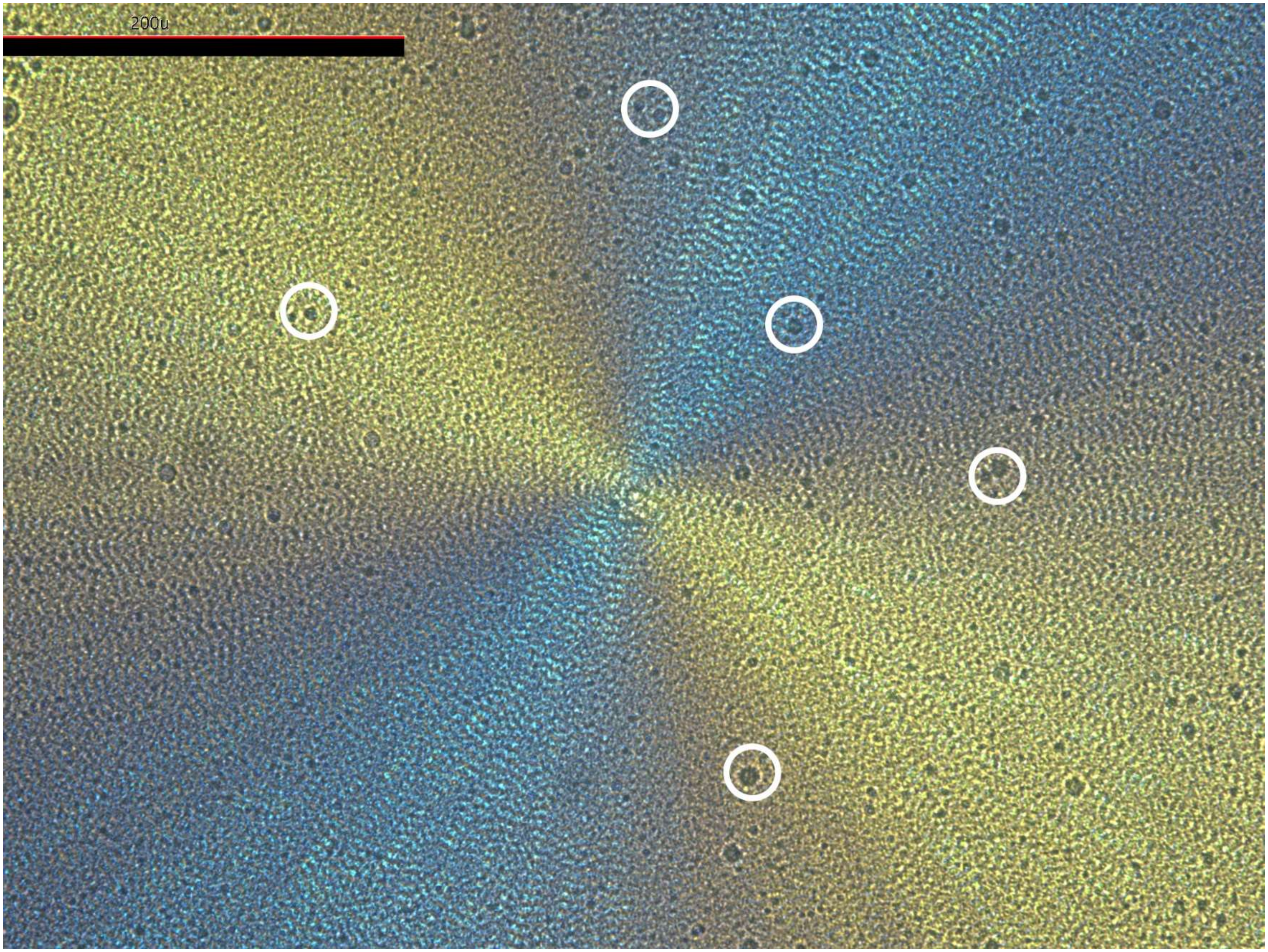

Figure S8. POM Image of PHB-*co*-HHx (7% HHx) loaded with 15% w/w DA spherulite (scale – 200 μm). White circles indicate some of the phase separated domains.

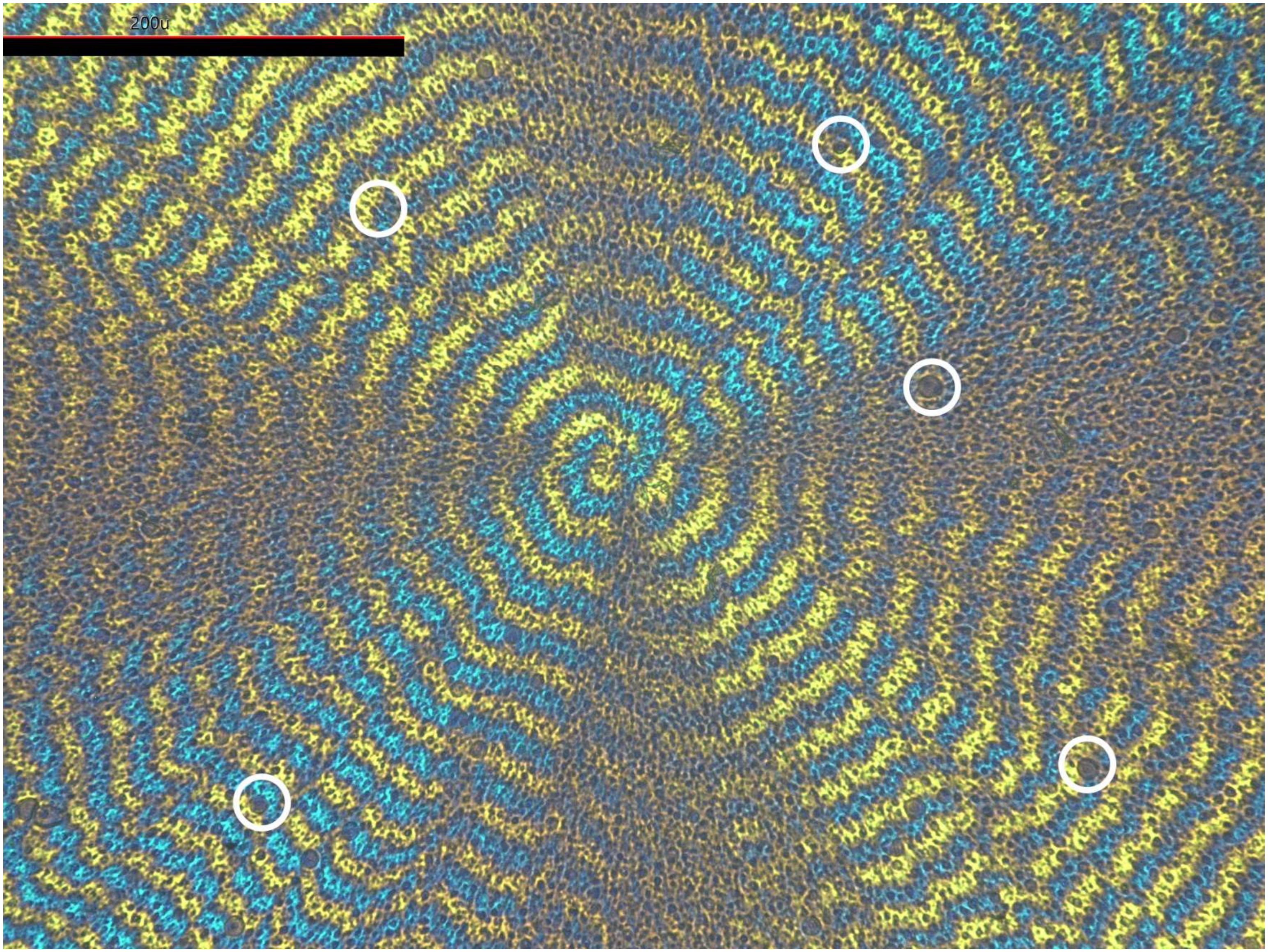

Figure S9. POM Image of PHB-*co*-HHx (7% HHx) loaded with 20% w/w DA spherulite (scale – 200 µm). White circles indicate some of the phase separated domains.

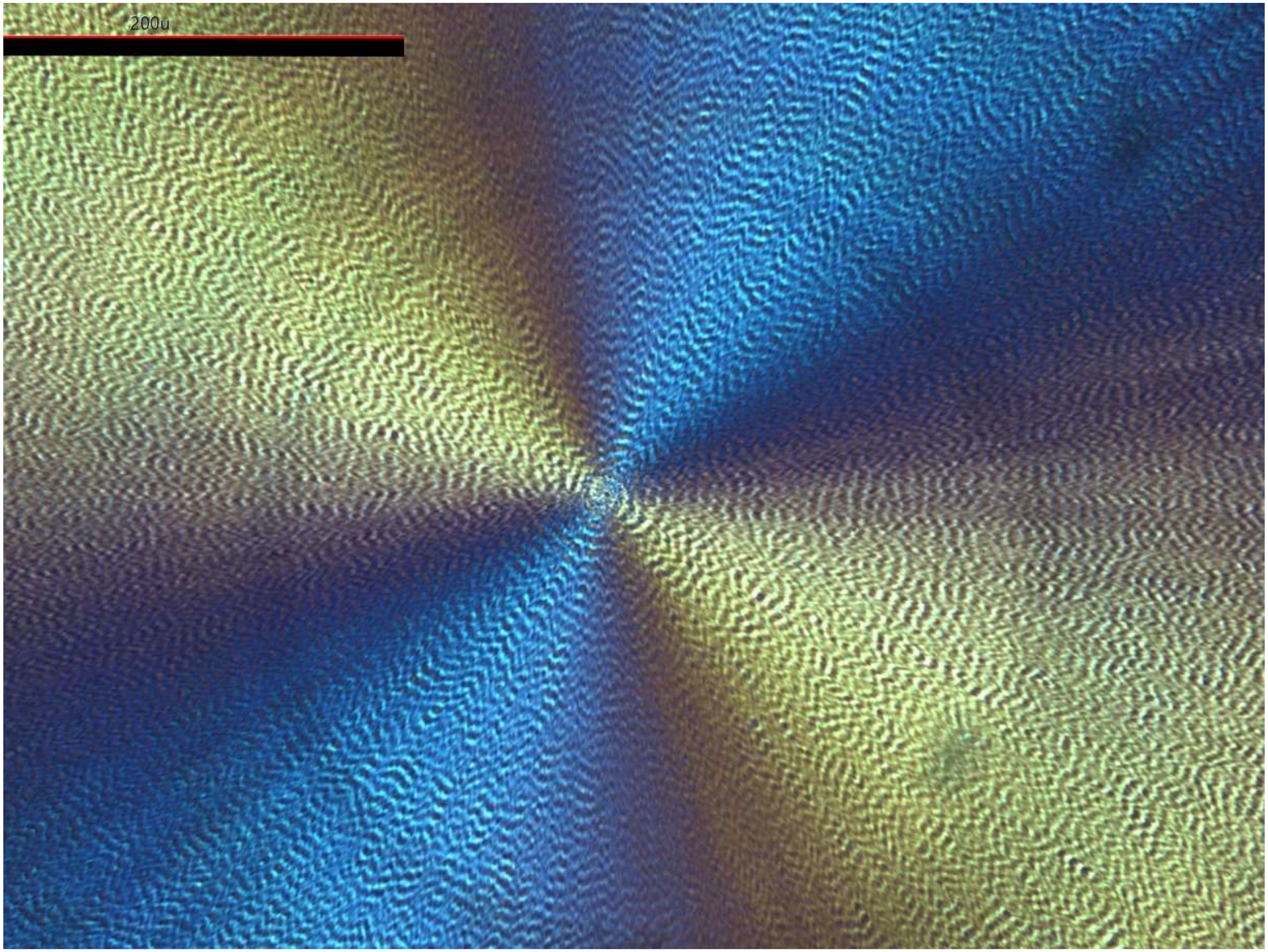

Figure S10. POM Image of PHB-*co*-HHx (7% HHx) loaded with 5% w/w EP spherulite (scale – 200 µm).

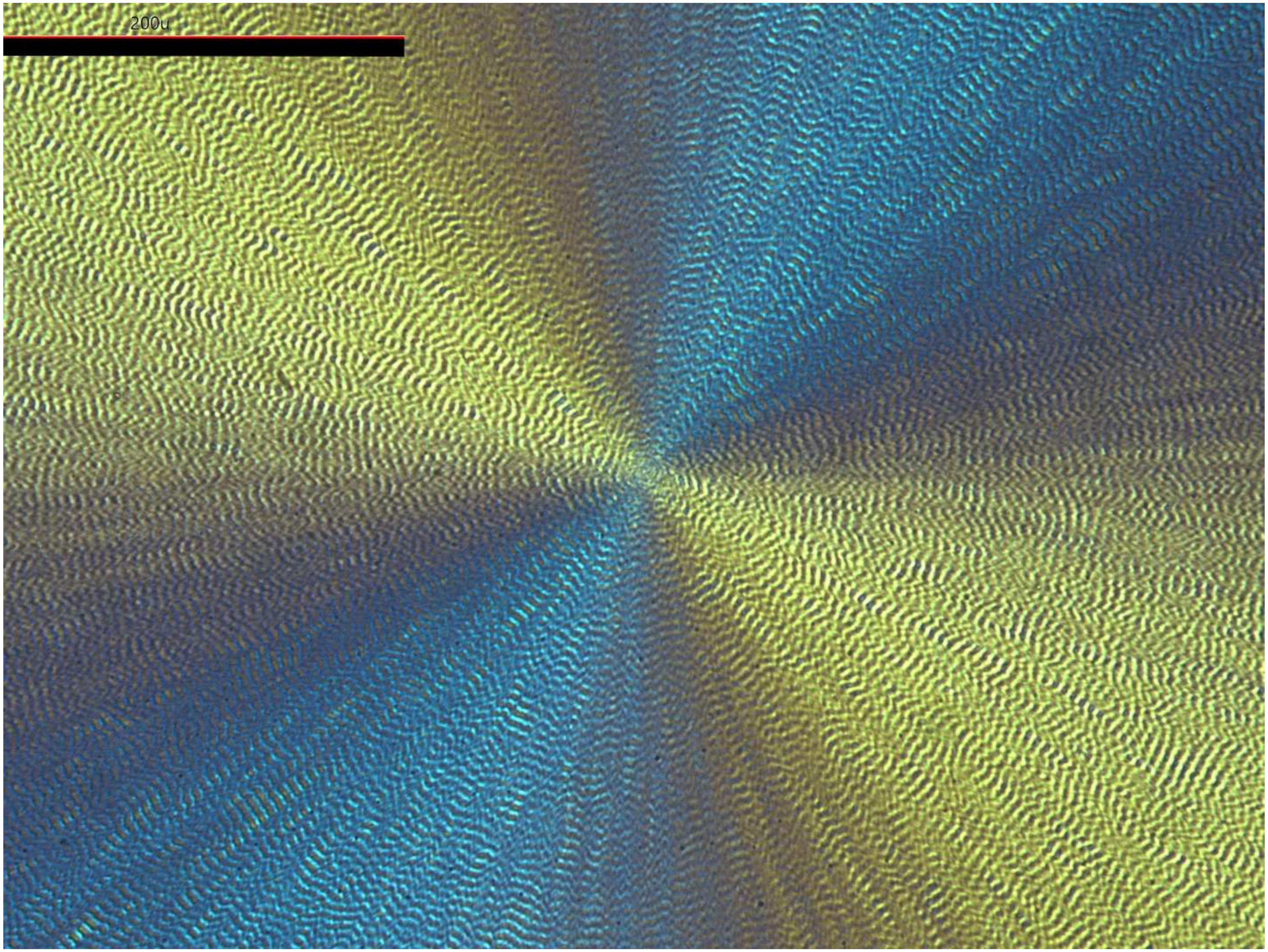

Figure S11. POM Image of PHB-*co*-HHx (7% HHx) loaded with 10% w/w EP spherulite (scale – 200 μm).

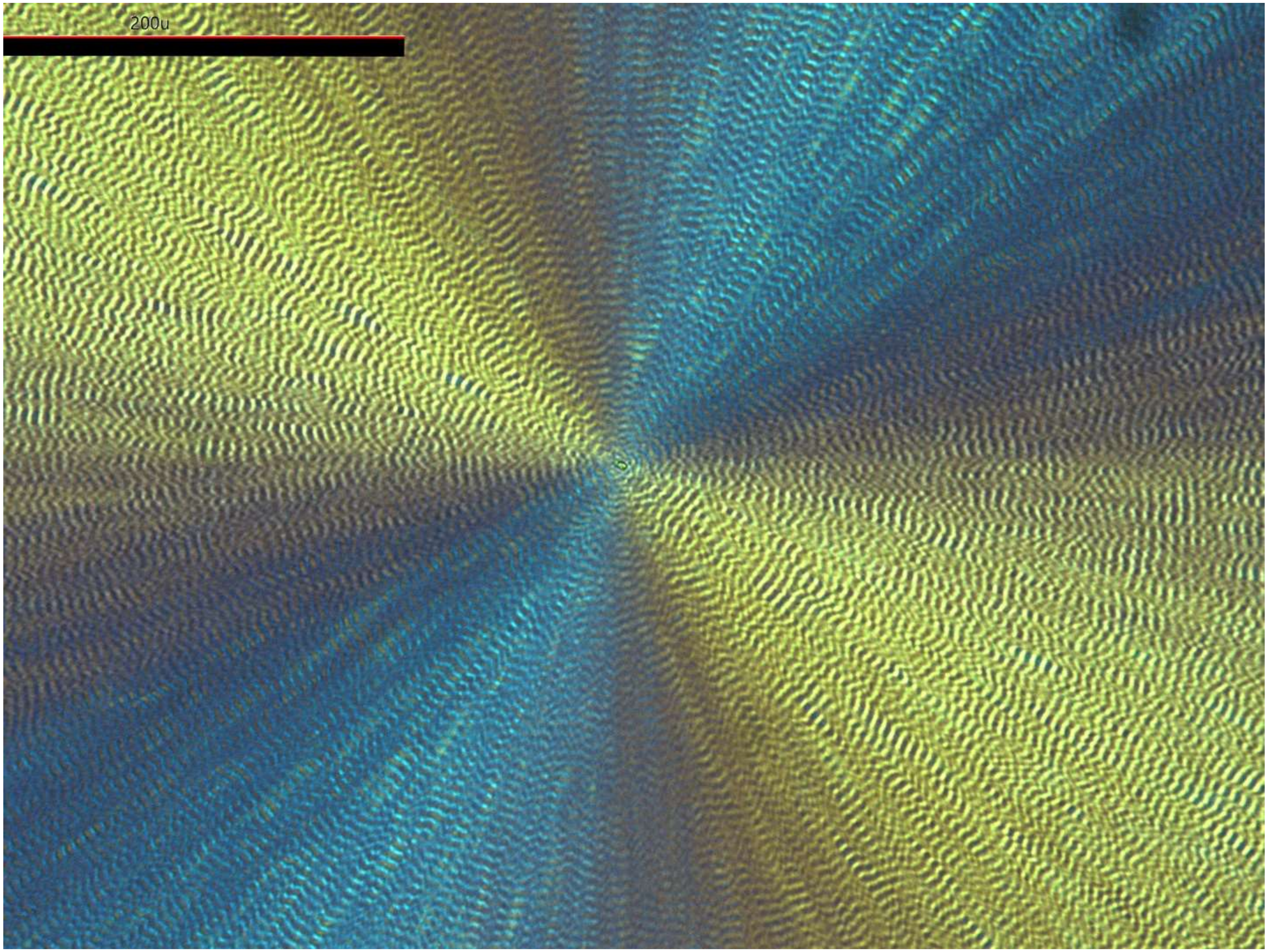

Figure S12. POM Image of PHB-*co*-HHx (7% HHx) loaded with 15% w/w EP spherulite (scale – 200 µm).

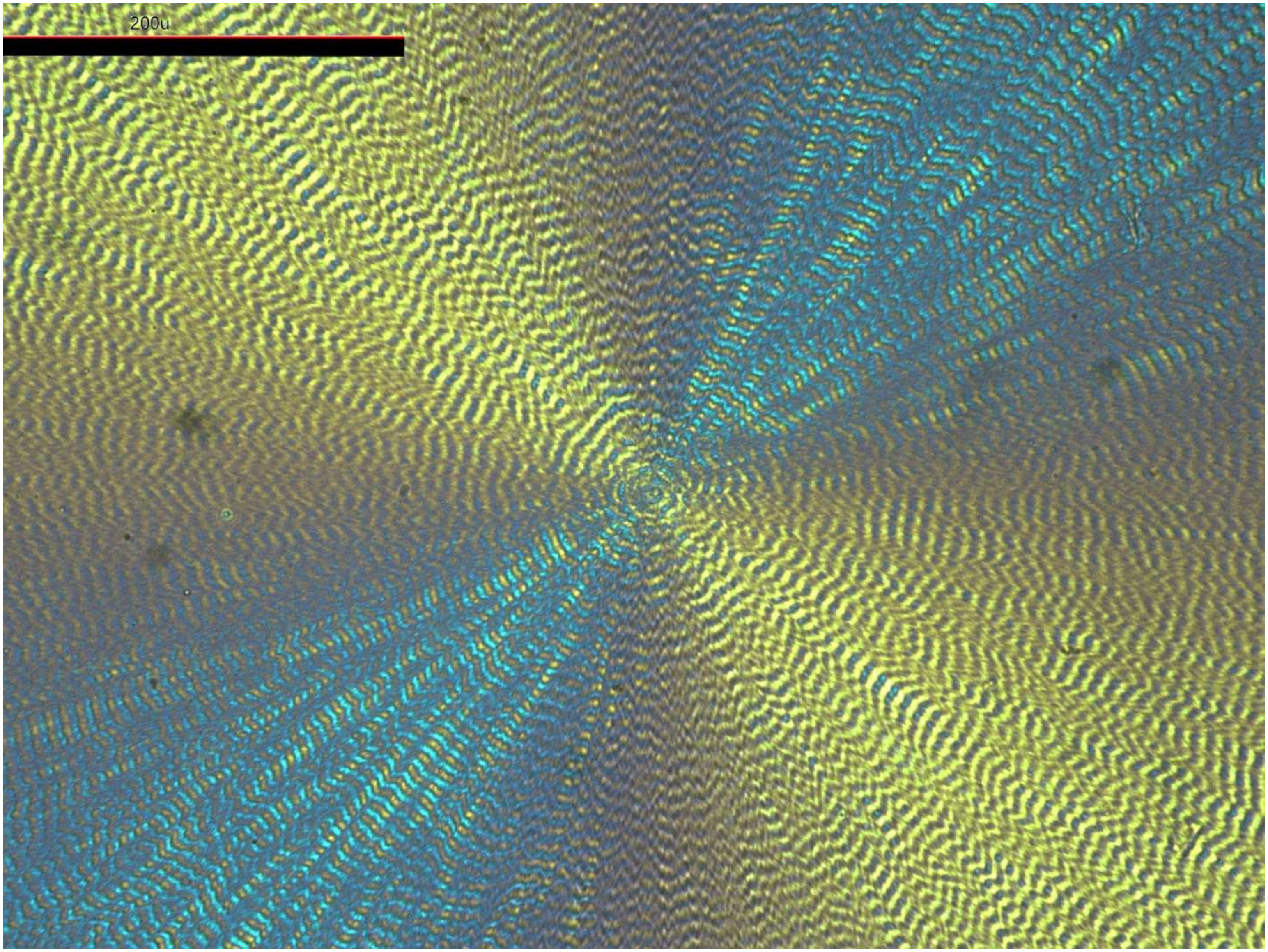

Figure S13. POM Image of PHB-*co*-HHx (7% HHx) loaded with 20% w/w EP spherulite (scale – 200 µm).

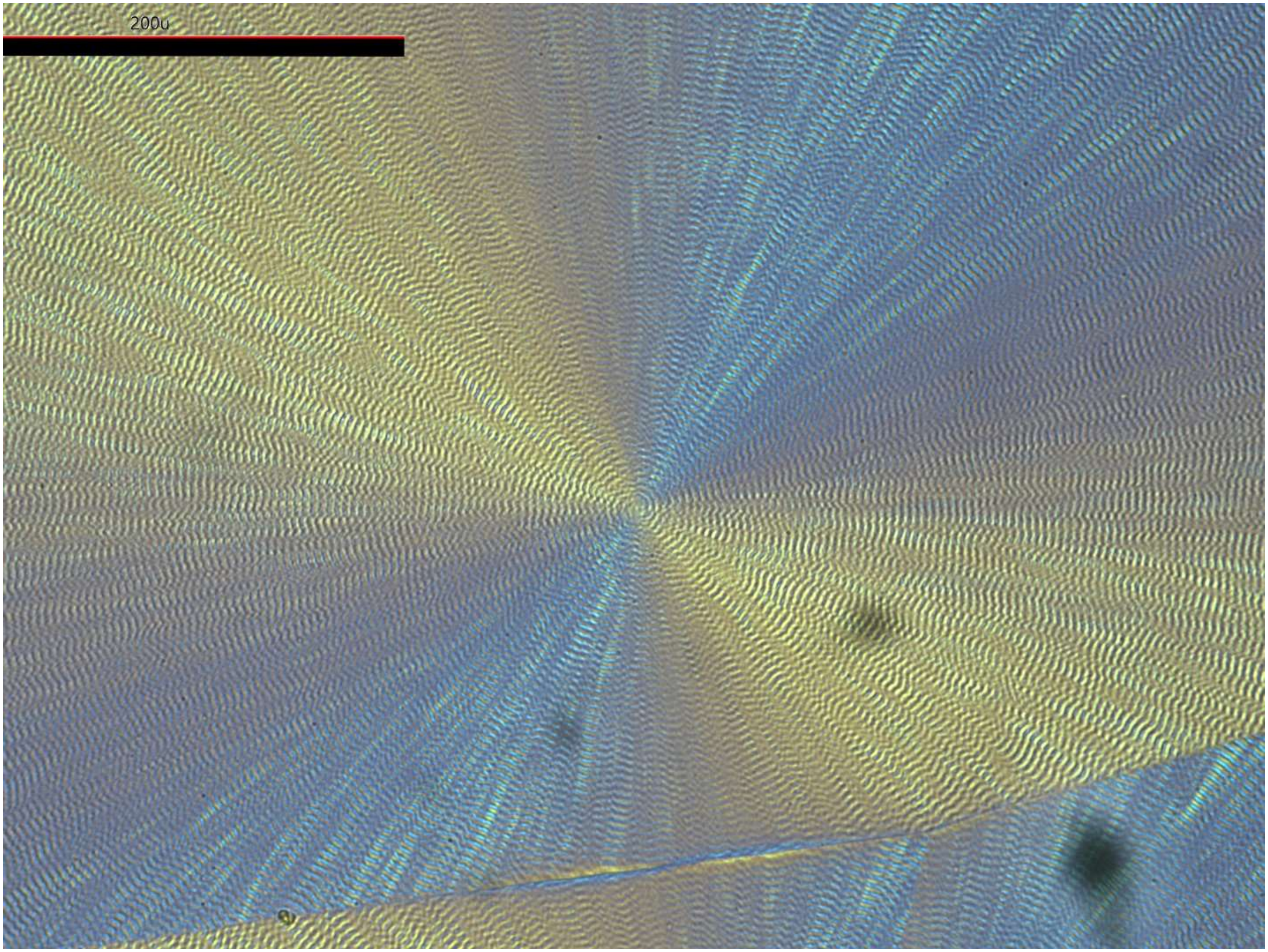

Figure S14. POM Image of PHB-*co*-HHx (7% HHx) loaded with 30% w/w EP spherulite (scale – 200 µm).

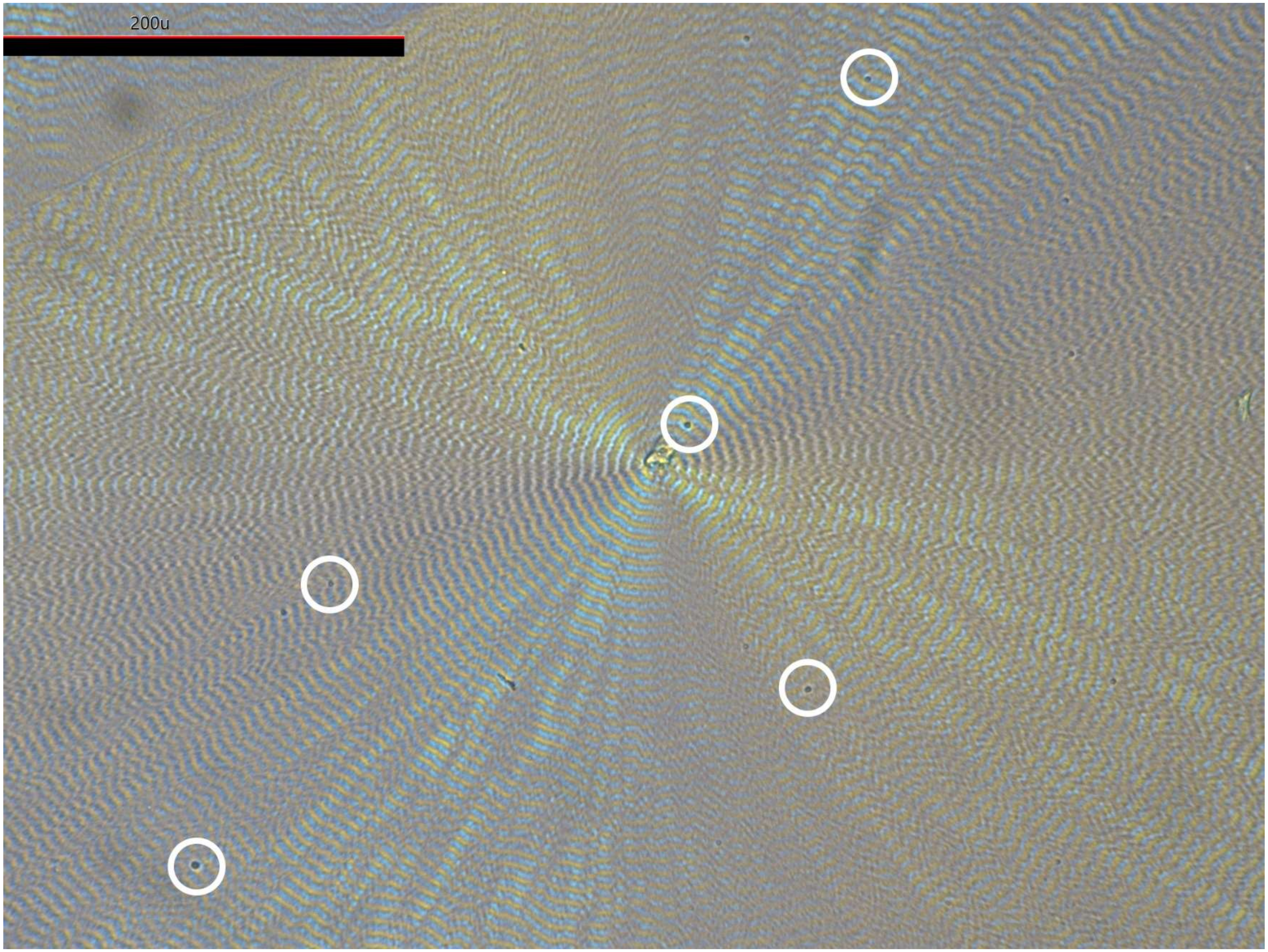

Figure S15. POM Image of PHB-*co*-HHx (7% HHx) loaded with 50% w/w EP spherulite (scale – 200 µm). White circles indicate some of the phase separated domains which appear as grainy texture on the spherulite surface.

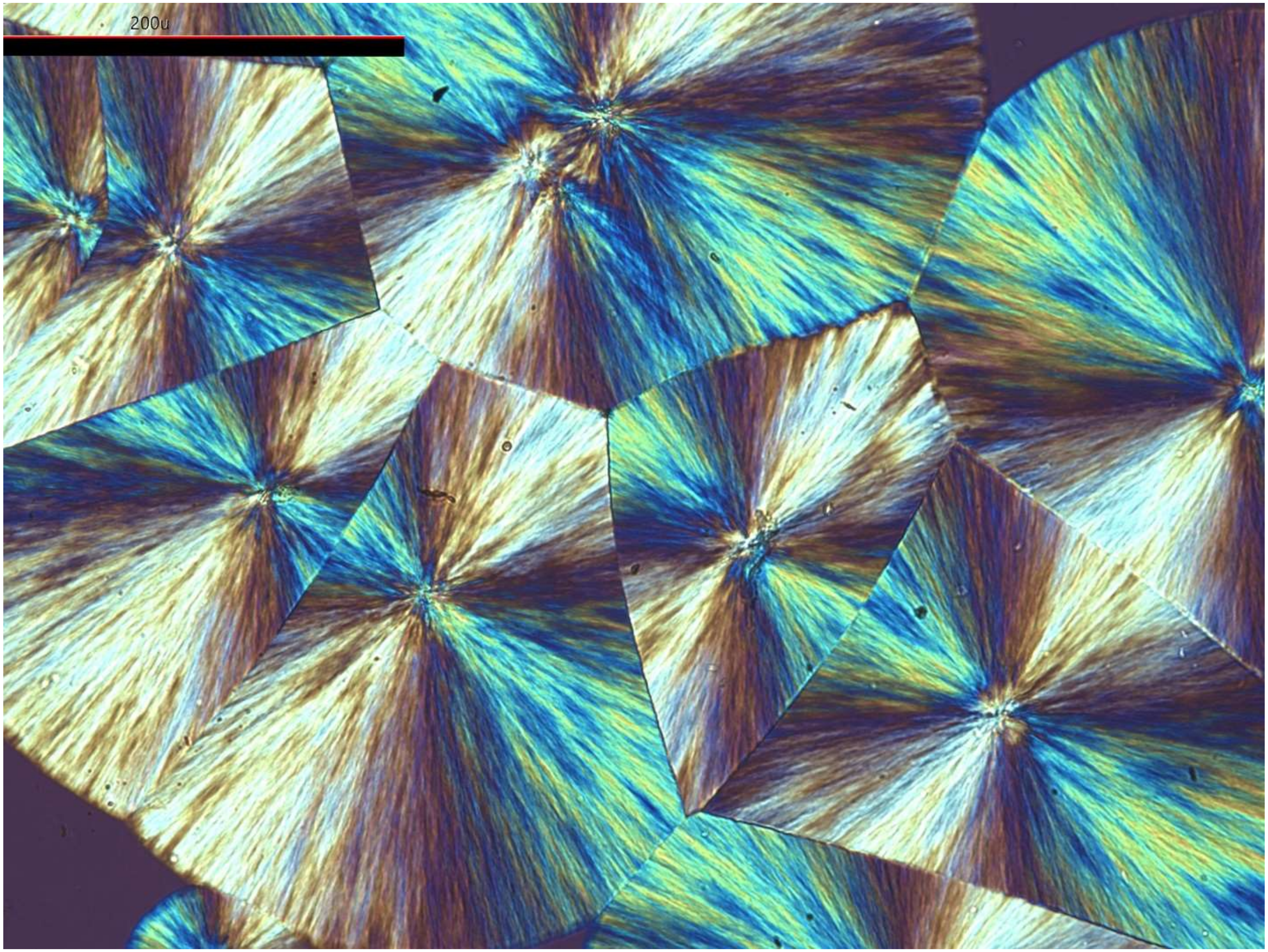

Figure S16. POM Image of PLA (Ingeo® 4032D) control spherulite (scale – 200 µm).

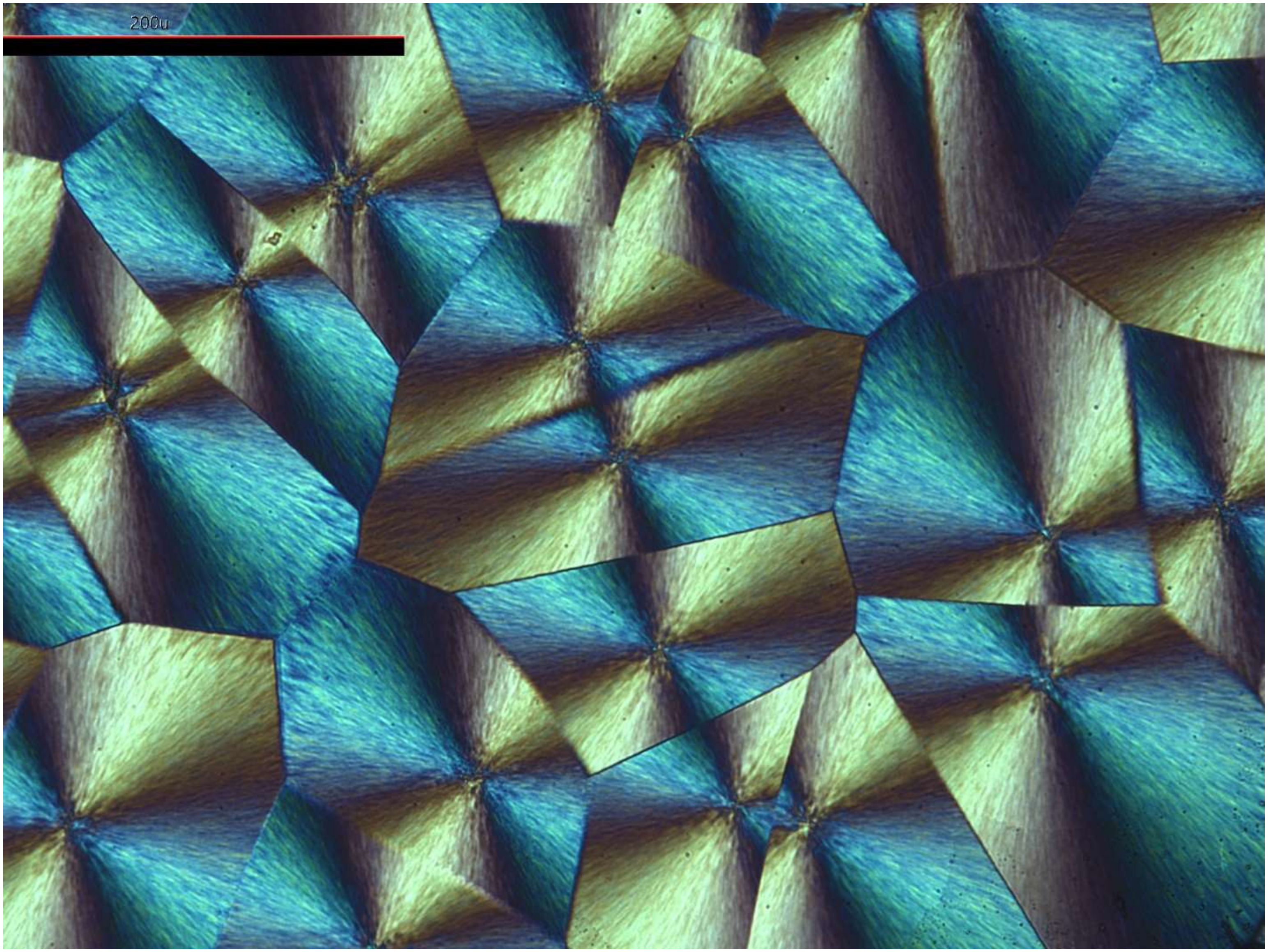

Figure S17. POM Image of PLA (Ingeo® 4032D) loaded with 2.5% DA spherulite (scale – 200 µm).

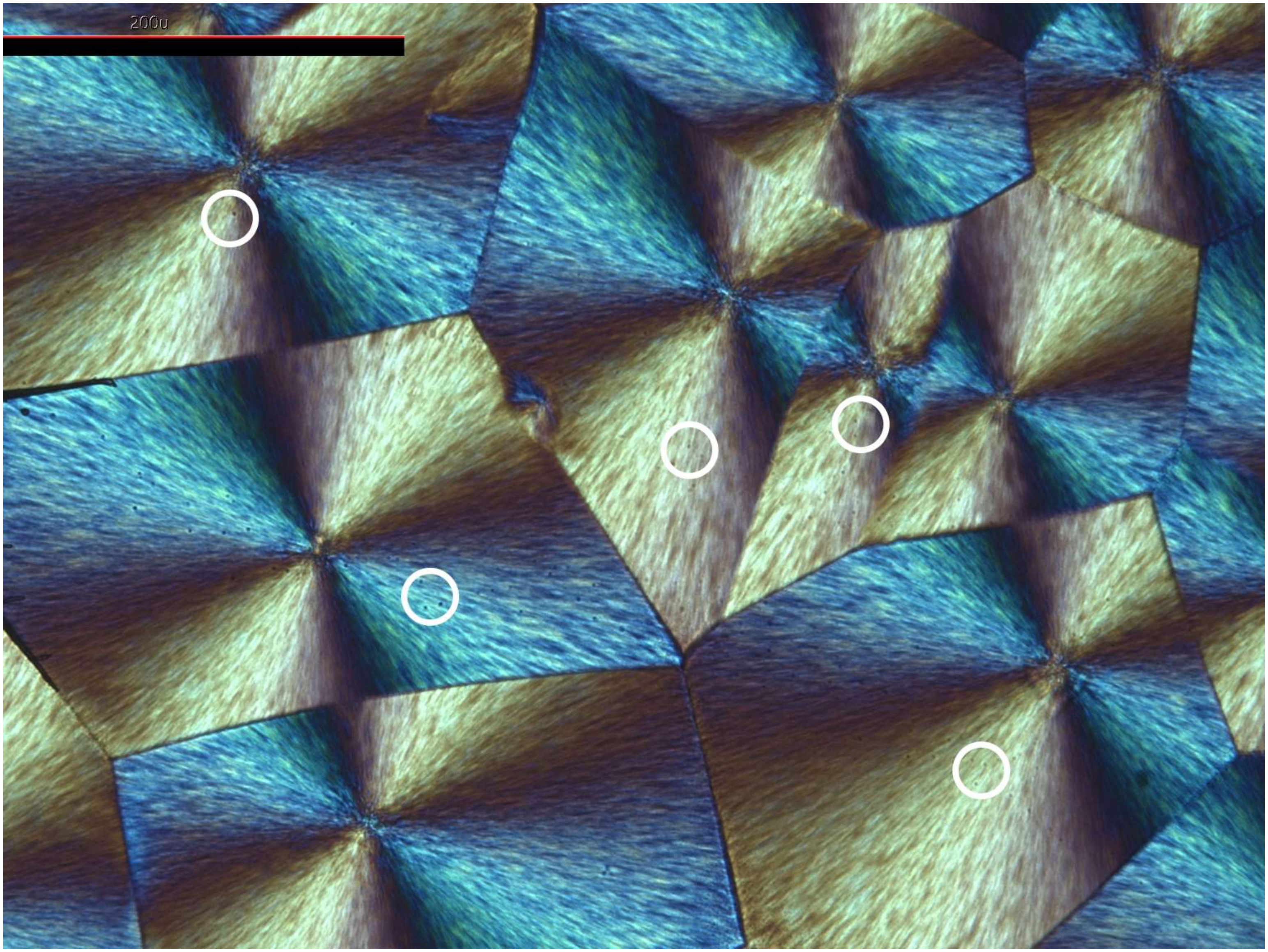

Figure S18. POM Image of PLA (Ingeo® 4032D) loaded with 5% DA spherulite (scale – 200 µm). White circles indicate some of the phase separated domains which appear as grainy texture on the spherulite surface.

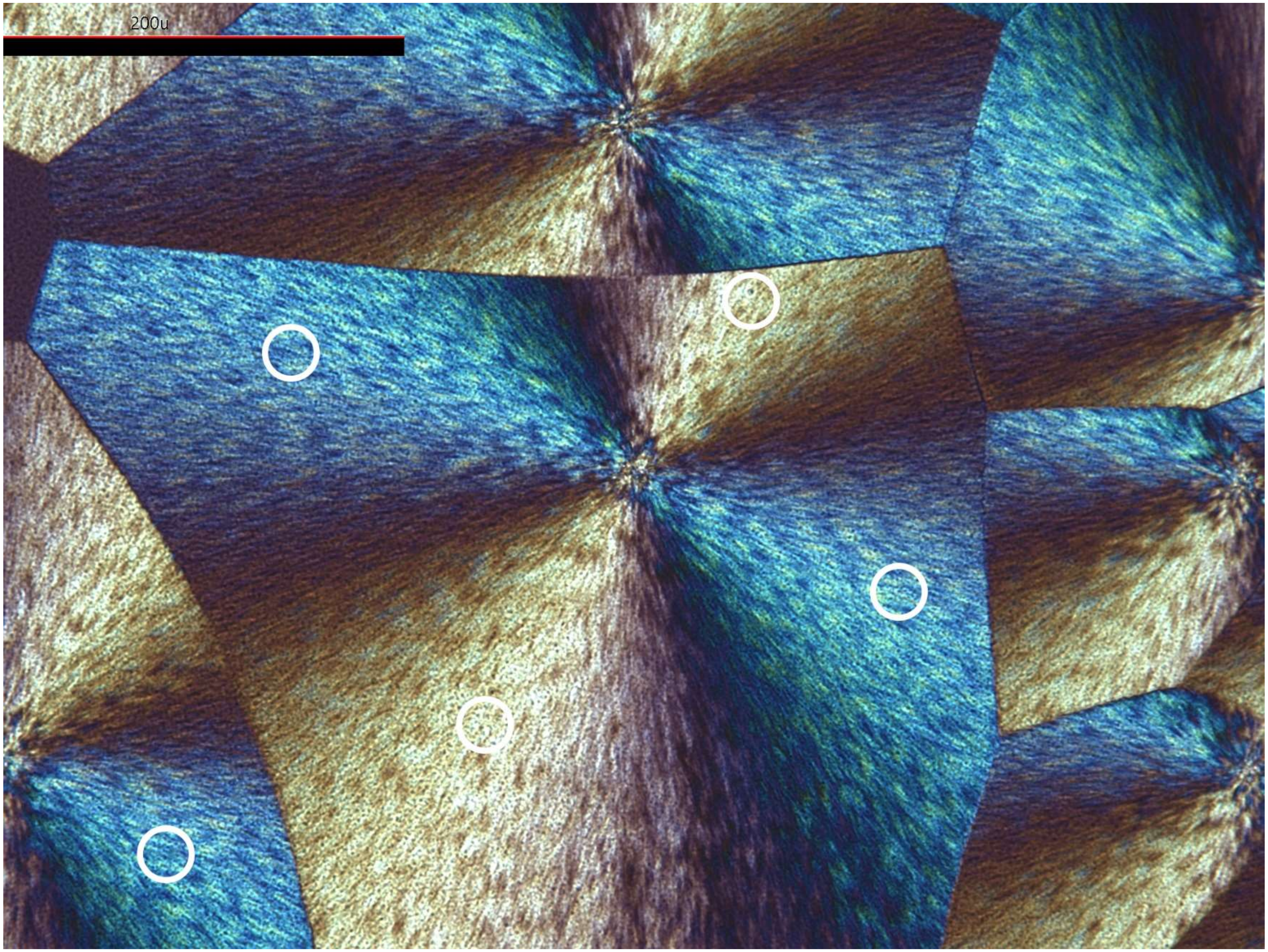

Figure S19. POM Image of PLA (Ingeo® 4032D) loaded with 10% DA spherulite (scale – 200 µm). White circles indicate some of the phase separated domains which appear as grainy texture on the spherulite surface.

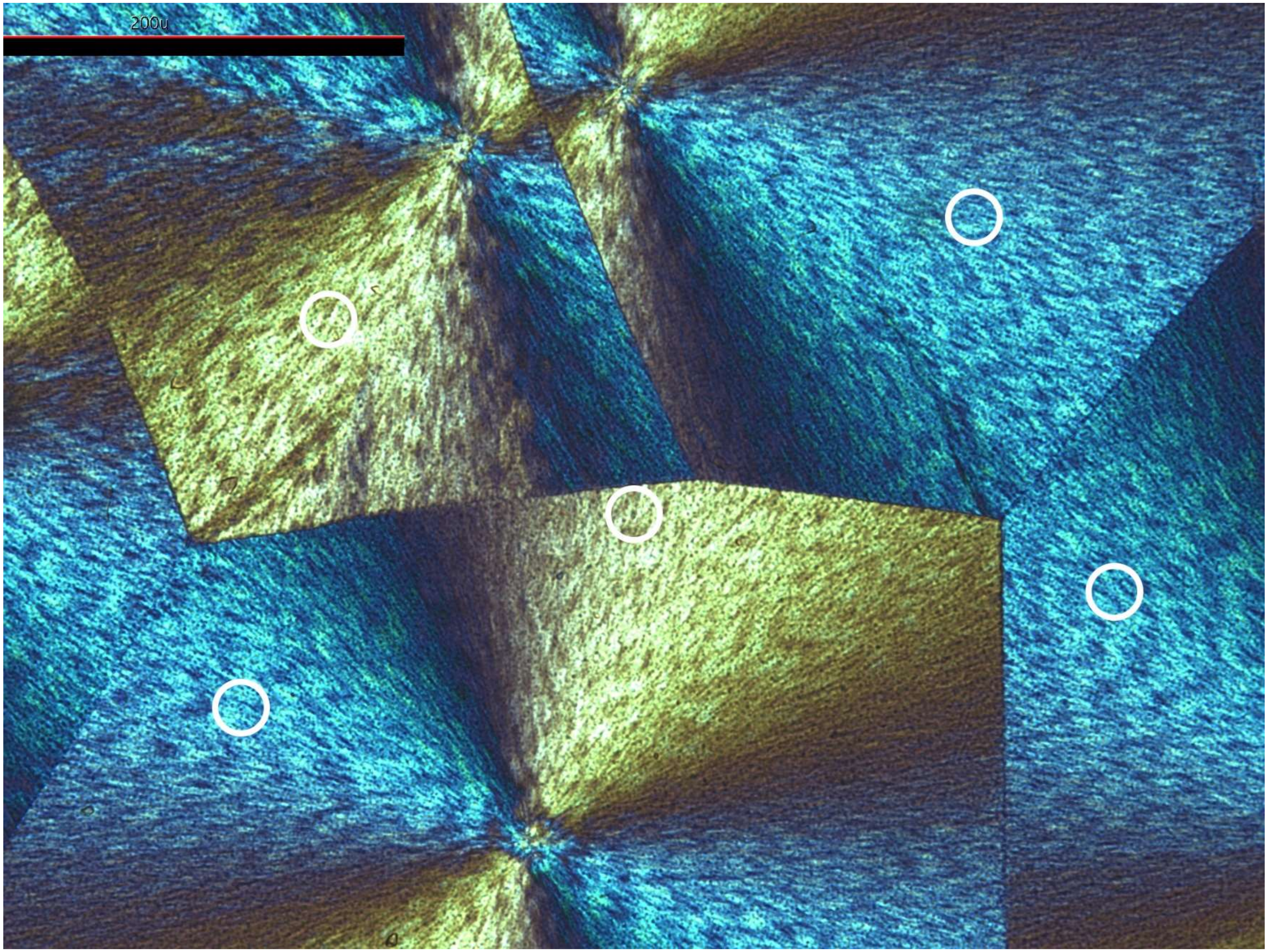

Figure S20. POM Image of PLA (Ingeo® 4032D) loaded with 15% DA spherulite (scale – 200 µm). White circles indicate some of the phase separated domains which appear as grainy texture on the spherulite surface.

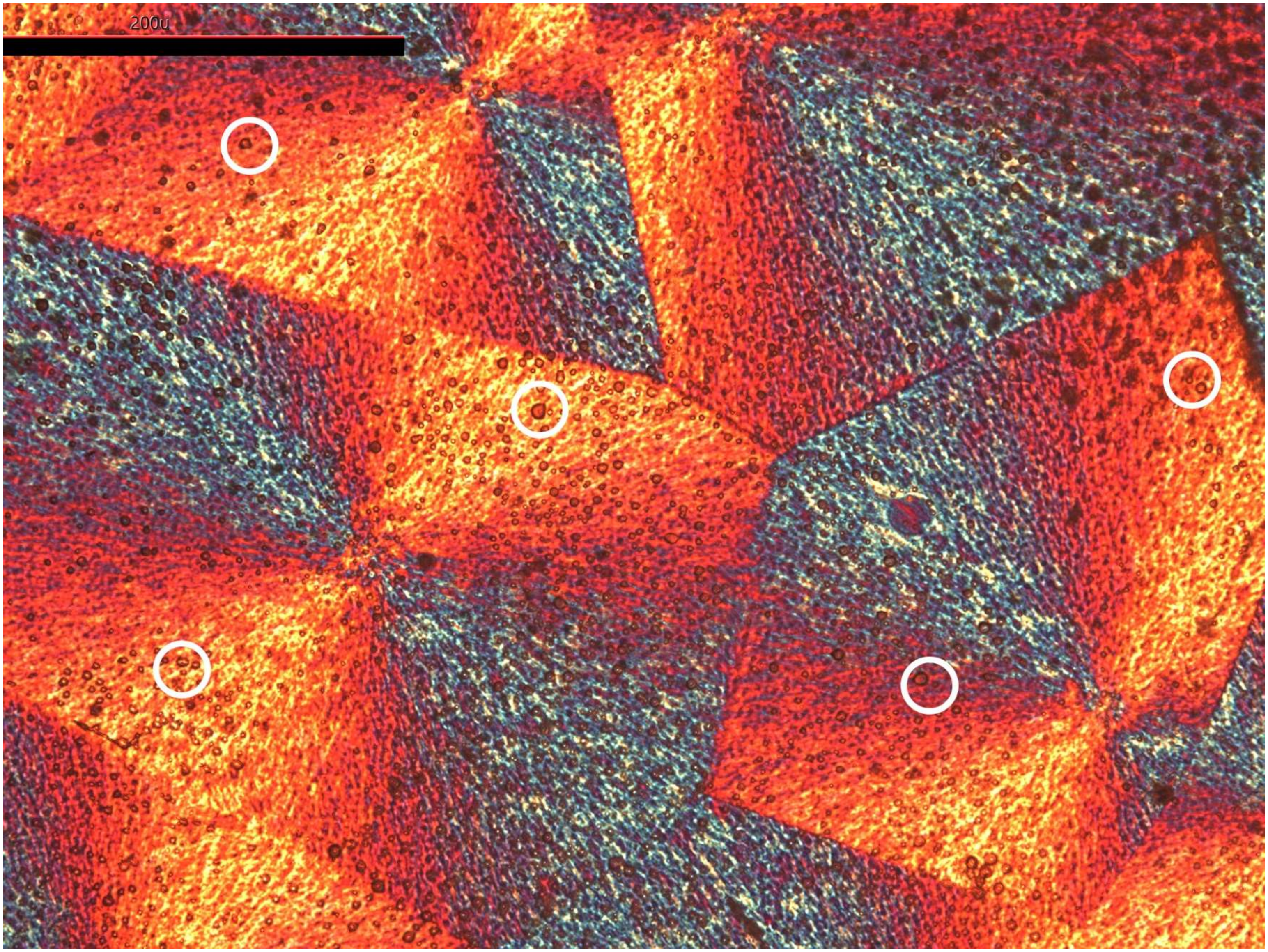

Figure S21. POM Image of PLA (Ingeo® 4032D) loaded with 20% DA spherulite (scale – 200 μm). White circles indicate some of the phase separated domains.

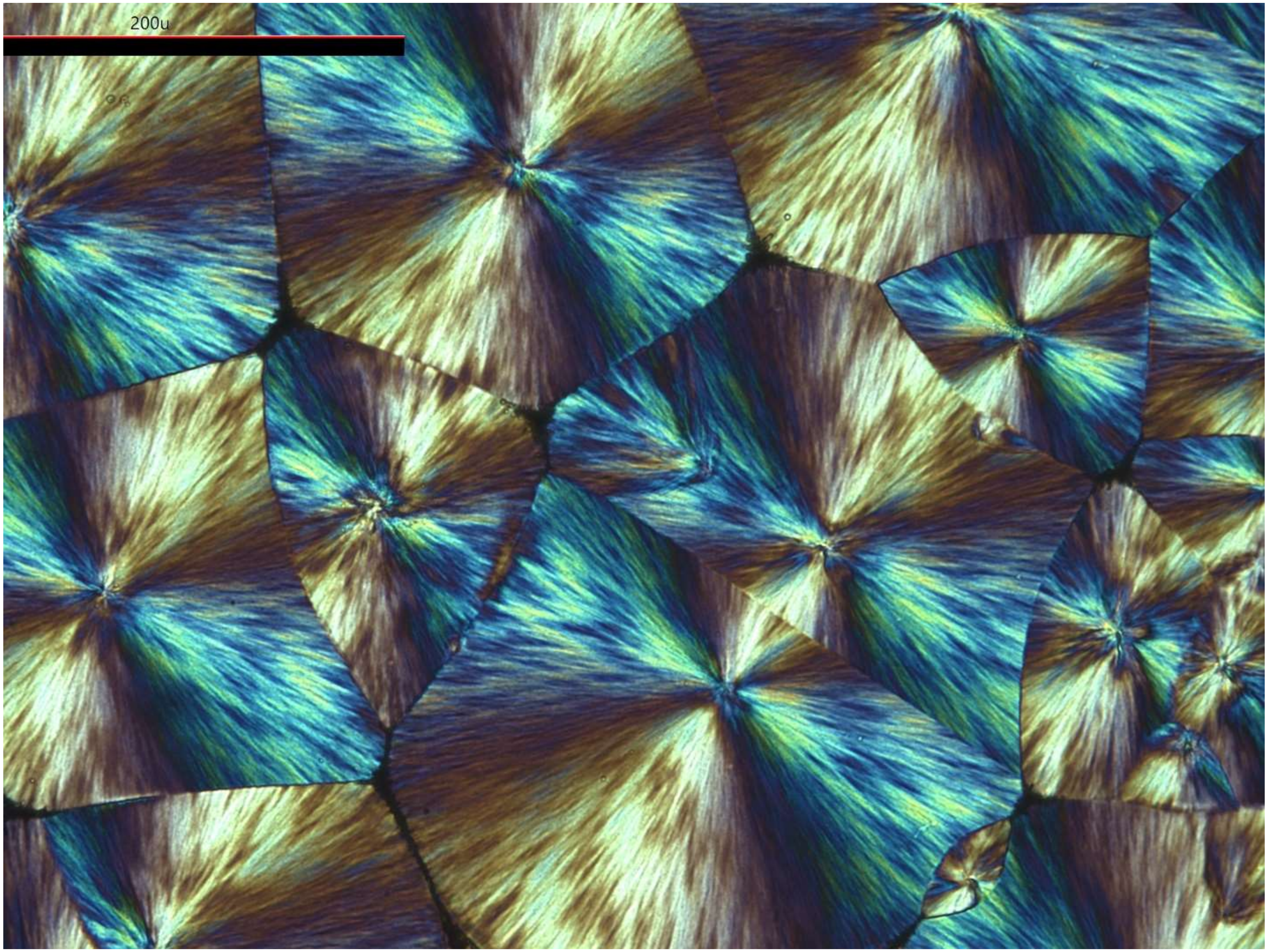

Figure S22. POM Image of PLA (Ingeo® 4032D) loaded with 5% EP spherulite (scale – 200 µm).

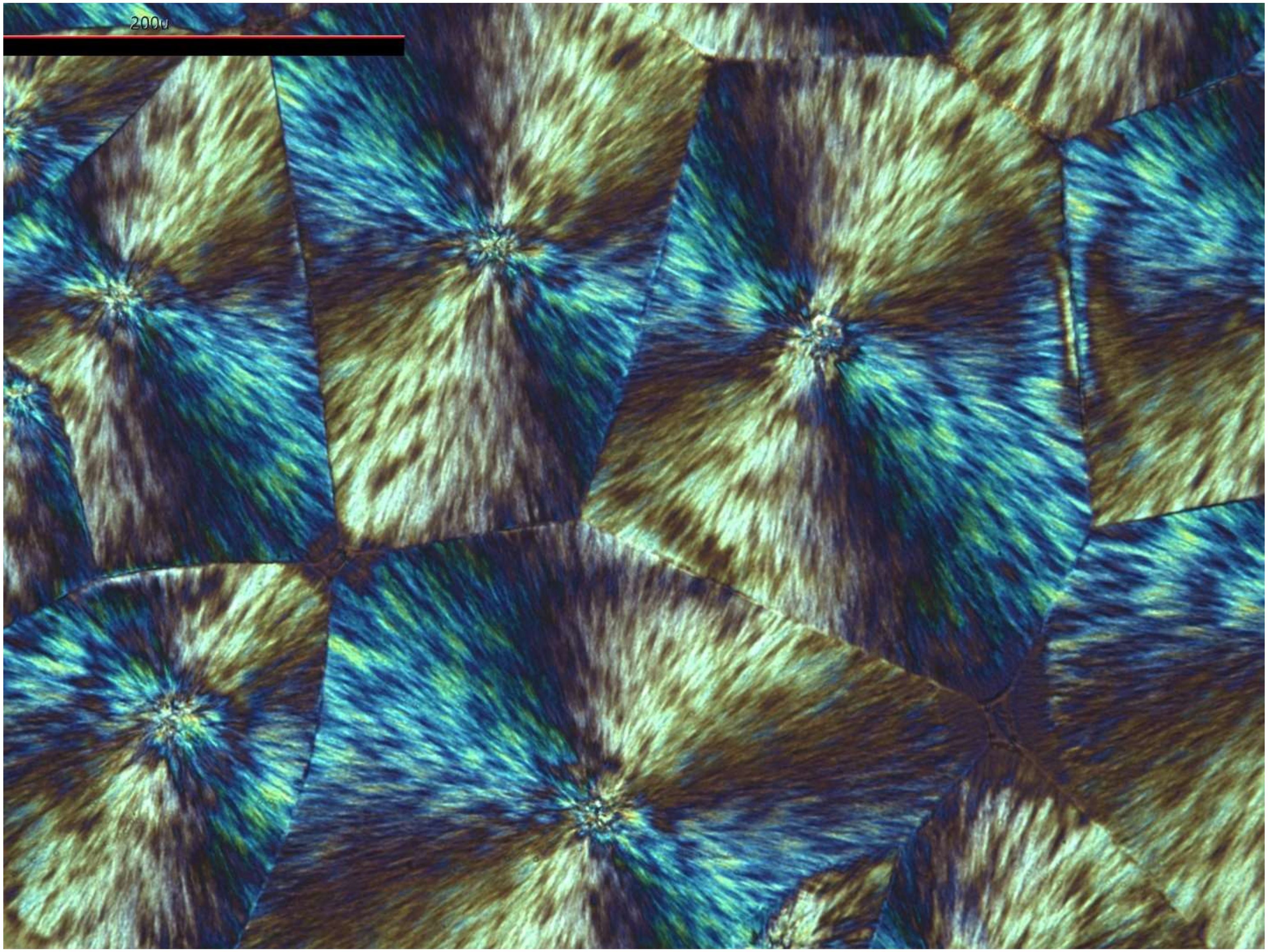

Figure S23. POM Image of PLA (Ingeo® 4032D) loaded with 10% EP spherulite (scale – 200 µm).

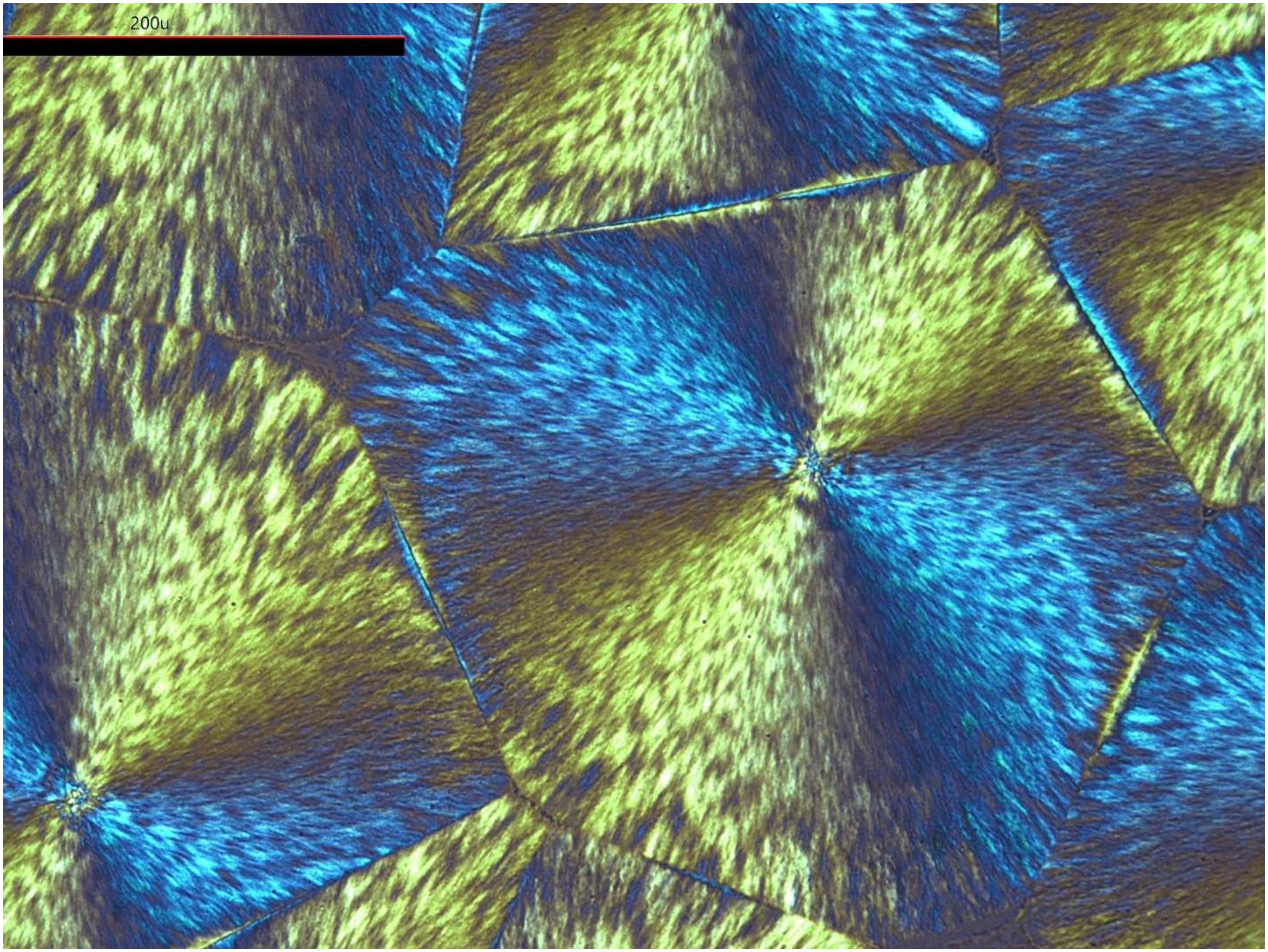

Figure S24. POM Image of PLA (Ingeo® 4032D) loaded with 15% EP spherulite (scale – 200 µm).

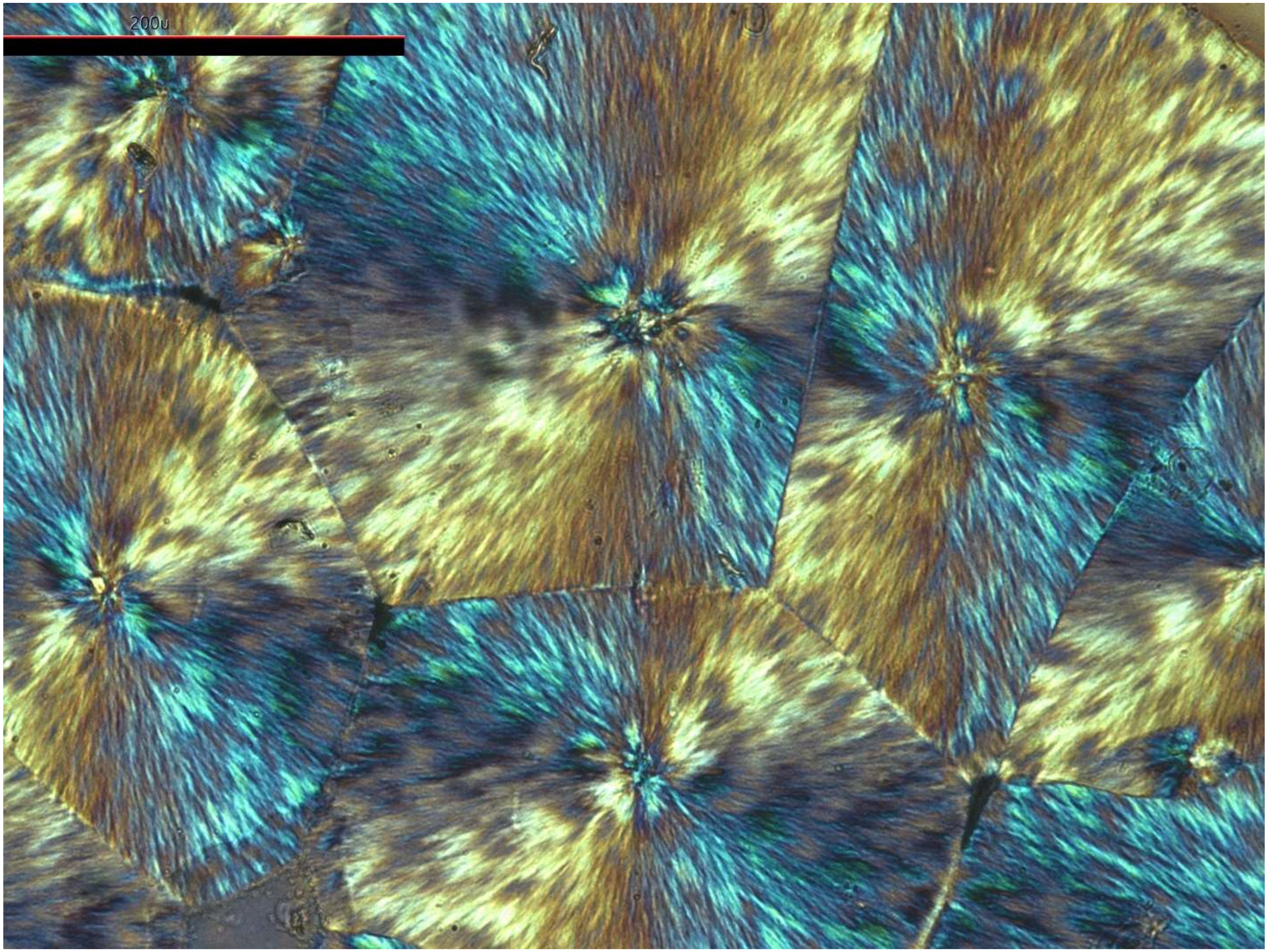

Figure S25. POM Image of PLA (Ingeo® 4032D) loaded with 20% EP spherulite (scale – 200 µm).

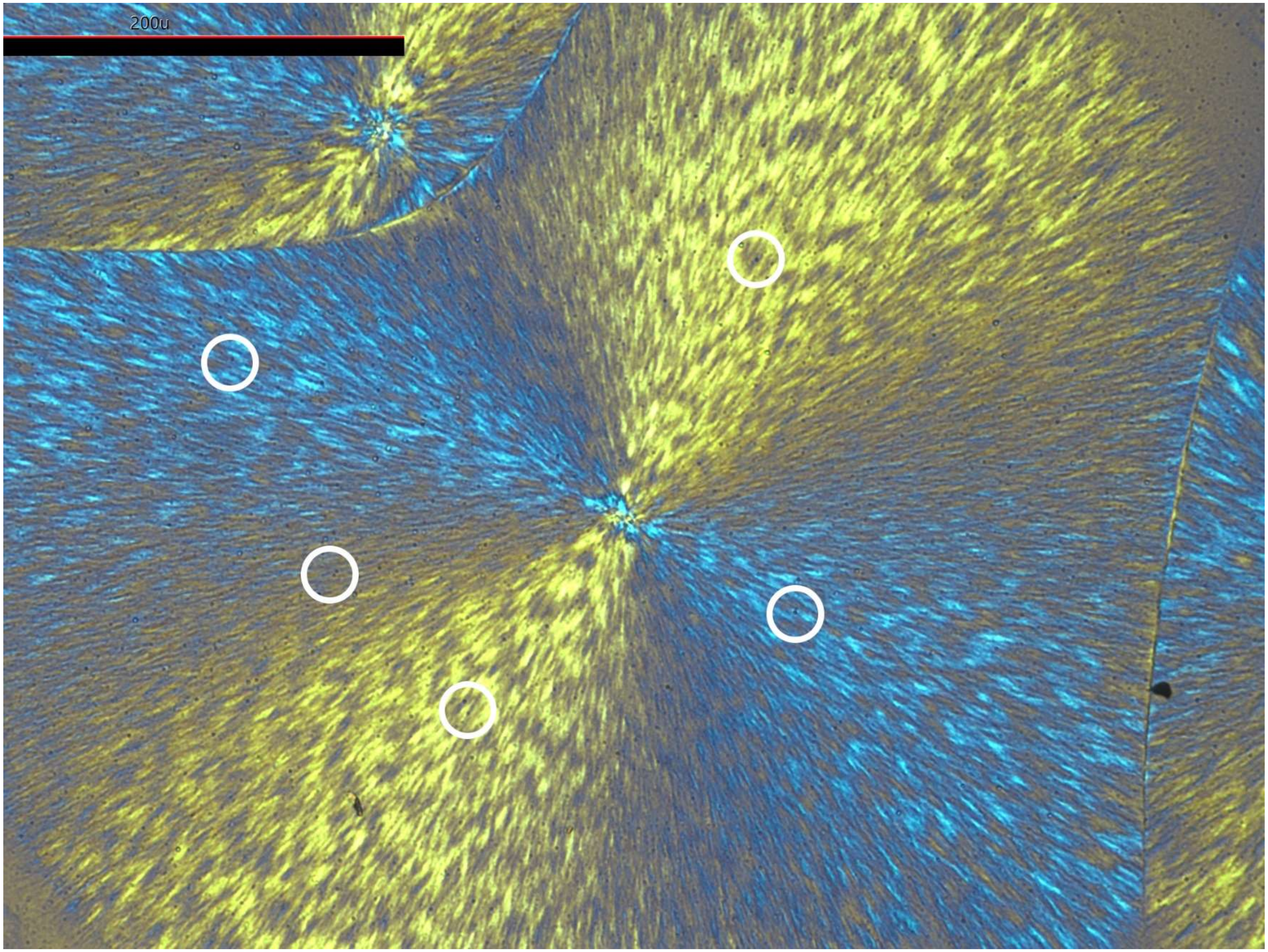

Figure S26. POM Image of PLA (Ingeo® 4032D) loaded with 30% EP spherulite (scale – 200 µm). White circles indicate some of the phase separated domains which appear as grainy texture on the spherulite surface.

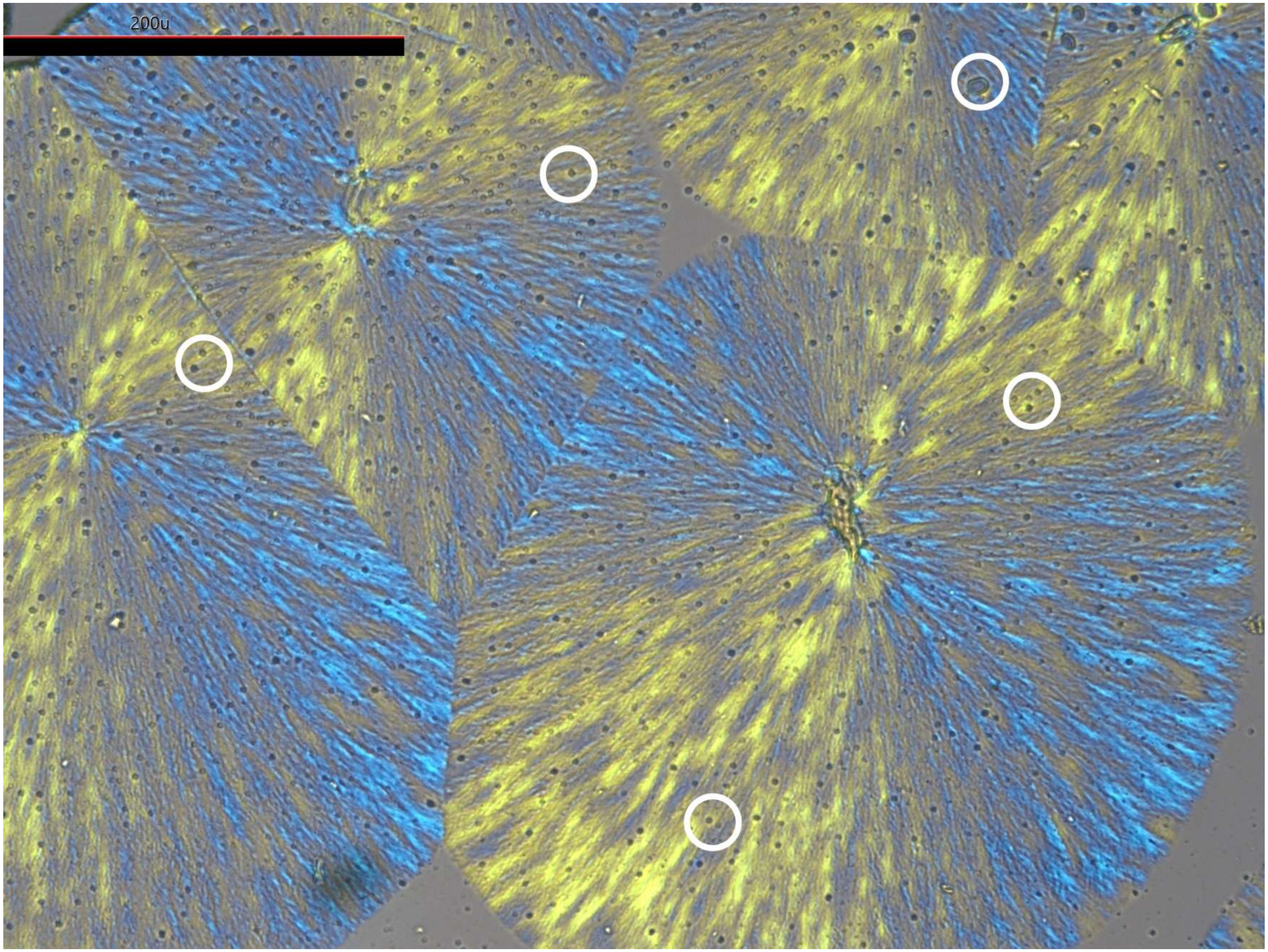

Figure S27. POM Image of PLA (Ingeo® 4032D) loaded with 50% EP spherulite (scale – 200 µm). White circles indicate some of the phase separated domains.

NMR Spectra

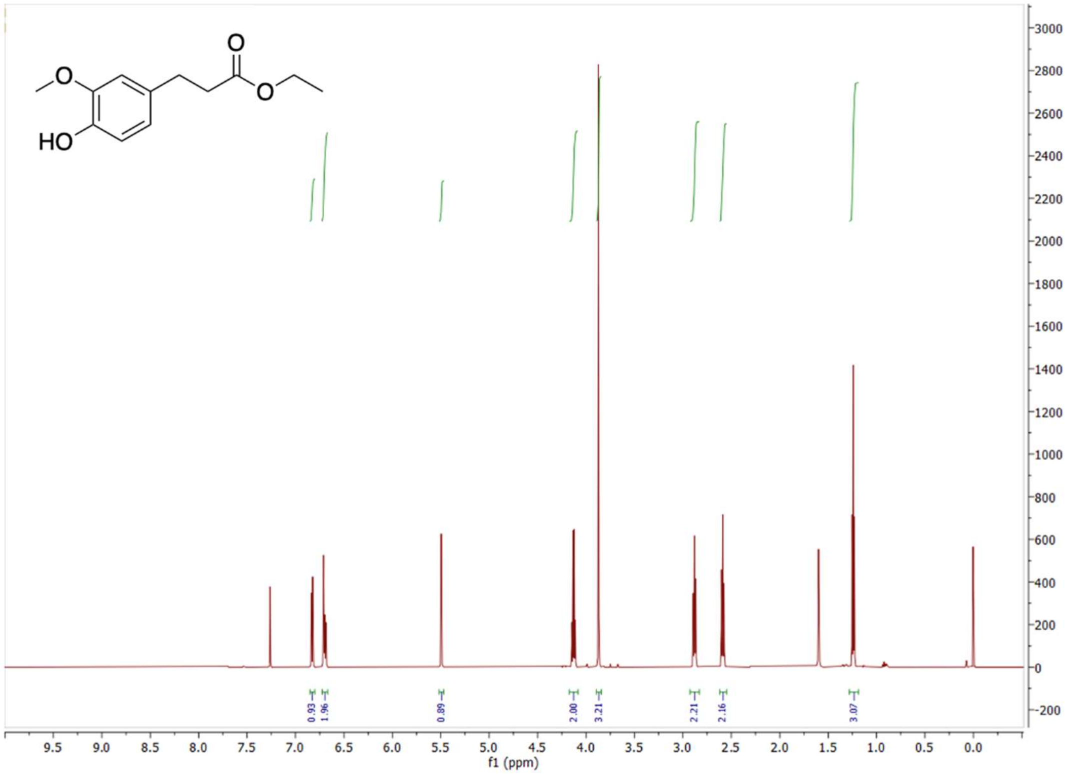

Figure S28. ^{1}H NMR spectrum of ethyl 3-(4-hydroxy-3-methoxyphenyl)propanoate.

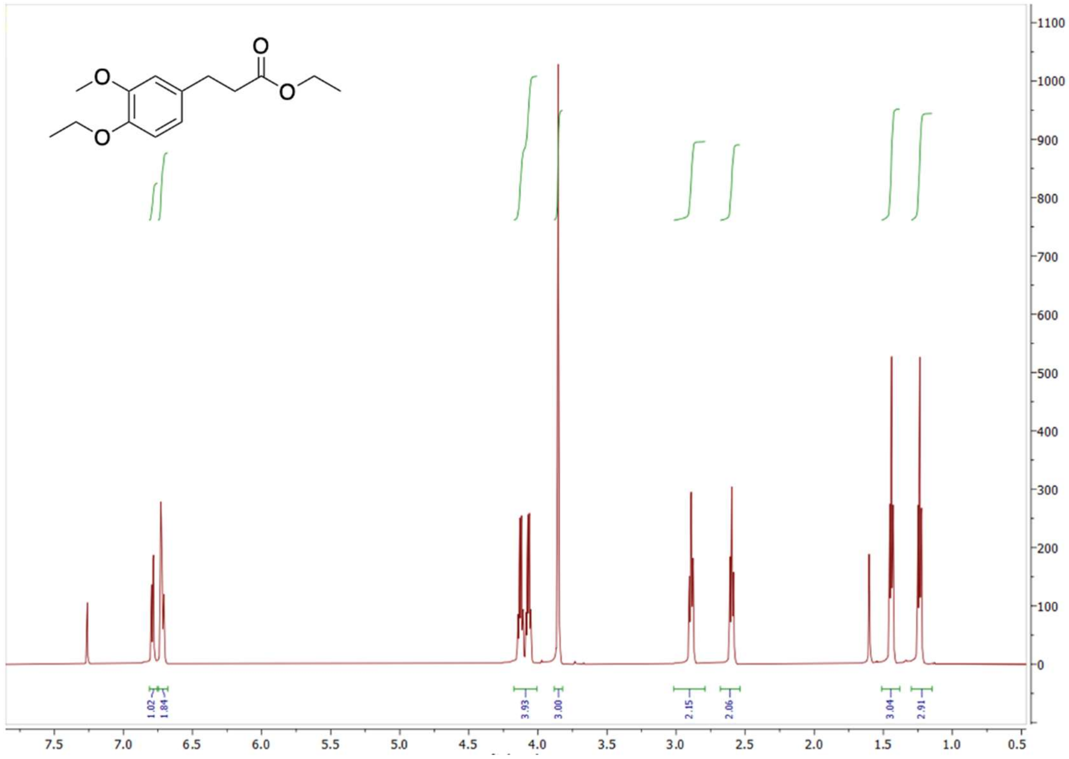

Figure S29. ^{1}H NMR spectrum of ethyl 3-(4-ethoxy-3-methoxyphenyl)propanoate (EP).

Nomenclature

Scoring Criteria

ImDis – Dissolution within 1 hour.

DisD – Dissolution over 24 hours.

DistT – Dissolution over the testing period (5 days).

Swl Unrec – Swollen but unrecoverable film (i.e., film collapses when disturbed).

Polymers

PHB-0HHx – Poly(3-hydroxybutyrate) (0 mol% HHx)

PHB-7HHx – Poly(3-hydroxybutyrate-*co*-3-hydroxyhexanoate) (7 mol% HHx)

PHB-13HHx – Poly(3-hydroxybutyrate-*co*-3-hydroxyhexanoate) (13 mol% HHx)

PHB-18HHx – Poly(3-hydroxybutyrate-*co*-3-hydroxyhexanoate) (18 mol% HHx)

PHB-30-4HB – P Poly(3-hydroxybutyrate-*co*-4-hydroxybutyrate) (>30 mol% 4HB)

PLA 4950D – Poly(lactic acid) (Natureworks Ingeo™ 4950D)

PLA 4060D – Poly(lactic acid) (Natureworks Ingeo™ 4060D)

PLA 4032D – Poly(lactic acid) (Natureworks Ingeo™ 4032D)

PLA 2500HP – Poly(lactic acid) (Natureworks Ingeo™ 2500HP)

PBS – Poly(butylene succinate) (BioPBS™ FZ91PM)

PBSA – Poly(butylene succinate-*co*-butylene adipate) (BioPBS™ FD92PM)

PBAT – Poly(butylene adipate-*co*-butylene terephthalate) (Ecoflex® F Blend C1200)

PCL – Poly(ε-caprolactone) (Capa® 6800D)

PTT – Poly(trimethylene terephthalate) (Sorona® Bright)

PEF – Poly(ethylene furanoate)

References

(1) AUSTRIA, T. V. *Doc Center*. 2023. https://www.tuv-at.be/green-marks/doc-center/ (accessed 2023 August, 23).

(2) Rosenboom, J.-G.; Langer, R.; Traverso, G. Bioplastics for a circular economy. *Nature Reviews Materials* **2022**, *7* (2), 117-137. DOI: 10.1038/s41578-021-00407-8.

(3) Winfield, D.; Ring, J.; Horn, J.; White, E. M.; Locklin, J. Semi-aromatic biobased polyesters derived from lignin and cyclic carbonates. *Green Chemistry* **2021**, *23* (23), 9658-9668. DOI: 10.1039/d1gc03135j.

(4) Laycock, B.; Halley, P.; Pratt, S.; Werker, A.; Lant, P. The chemomechanical properties of microbial polyhydroxyalkanoates. *Progress in Polymer Science* **2013**, *38* (3-4), 536-583. DOI: 10.1016/j.progpolymsci.2012.06.003.

(5) Garlotta, D. *Journal of Polymers and the Environment* **2001**, *9* (2), 63-84. DOI: 10.1023/a:1020200822435.

(6) Miyata, T.; Masuko, T. Crystallization behaviour of poly(tetramethylene succinate). *Polymer* **1998**, *39* (6-7), 1399-1404. DOI: 10.1016/s0032-3861(97)00418-7.

(7) Jiang, G.; Yu, L. High Strength and Barrier Properties of Biodegradable PPC/PBSA Blends Prepared by Reaction Compatibilization for Promising Application in Packaging. *Macromolecular Materials and Engineering* **2021**, *306* (7). DOI: 10.1002/mame.202000723.

(8) Mohanty, S.; Nayak, S. K. Aromatic-aliphatic poly(butylene adipate-co-terephthalate) bionanocomposite: Influence of organic modification on structure and properties. *Polymer Composites* **2009**, NA-NA. DOI: 10.1002/pc.20906.

(9) Crescenzi, V.; Manzini, G.; Calzolari, G.; Borri, C. Thermodynamics of fusion of poly-β-propiolactone and poly-ϵ-caprolactone. comparative analysis of the melting of aliphatic polylactone and polyester chains. *European Polymer Journal* **1972**, *8* (3), 449-463. DOI: 10.1016/0014-3057(72)90109-7.

(10) Huang, J.-M.; Chang, F.-C. Crystallization kinetics of poly(trimethylene terephthalate). *Journal of Polymer Science Part B: Polymer Physics* **2000**, *38* (7), 934-941. DOI: 10.1002/(sici)1099-0488(20000401)38:7<934::Aid-polb4>3.0.Co;2-r.

(11) Papageorgiou, G. Z.; Tsanaktsis, V.; Bikiaris, D. N. Synthesis of poly(ethylene furandicarboxylate) polyester using monomers derived from renewable resources: thermal behavior comparison with PET and PEN. *Phys. Chem. Chem. Phys.* **2014**, *16* (17), 7946-7958. DOI: 10.1039/c4cp00518j.
